# Macromolecularly crowded *in vitro* microenvironments accelerate the production of extracellular matrix-rich supramolecular assemblies

**DOI:** 10.1038/srep08729

**Published:** 2015-03-04

**Authors:** Pramod Kumar, Abhigyan Satyam, Xingliang Fan, Estelle Collin, Yury Rochev, Brian J. Rodriguez, Alexander Gorelov, Simon Dillon, Lokesh Joshi, Michael Raghunath, Abhay Pandit, Dimitrios I. Zeugolis

**Affiliations:** 1 Network of Excellence for Functional Biomaterials (NFB), National University of Ireland Galway (NUI Galway), Bioscience Research Building, Galway, Ireland; 2 Conway Institute of Biomolecular & Biomedical Research, University College Dublin, Dublin, Ireland; 3 School of Chemistry & Chemical Biology, University College Dublin, Dublin, Ireland; 4 BIDMC Genomics, Proteomics, Bioinformatics and Systems Biology Center, Beth Israel Deaconess Medical Center, Harvard Medical School, Boston, USA; 5 Alimentary Glycoscience Research Cluster, NUI Galway, Galway, Ireland; 6 Department of Bioengineering, Faculty of Engineering, National University of Singapore Tissue Engineering Programme, Department of Biochemistry, Yong Loo Lin School of Medicine, National University of Singapore, Singapore

## Abstract

Therapeutic strategies based on the principles of tissue engineering by self-assembly put forward the notion that functional regeneration can be achieved by utilising the inherent capacity of cells to create highly sophisticated supramolecular assemblies. However, in dilute *ex vivo* microenvironments, prolonged culture time is required to develop an extracellular matrix-rich implantable device. Herein, we assessed the influence of macromolecular crowding, a biophysical phenomenon that regulates intra- and extra-cellular activities in multicellular organisms, in human corneal fibroblast culture. In the presence of macromolecules, abundant extracellular matrix deposition was evidenced as fast as 48 h in culture, even at low serum concentration. Temperature responsive copolymers allowed the detachment of dense and cohesive supramolecularly assembled living substitutes within 6 days in culture. Morphological, histological, gene and protein analysis assays demonstrated maintenance of tissue-specific function. Macromolecular crowding opens new avenues for a more rational design in engineering of clinically relevant tissue modules *in vitro*.

Tissue engineering by self-assembly^1^,^2^, cell-sheet tissue engineering^3^, scaffold-free tissue engineering^4^ or modular tissue engineering^5^,^6^ utilise nature's sophistication to create bottom up supramolecular assemblies. The rationale of either of these approaches lays on the fact that cells are the traditional extracellular matrix (ECM) builders and create tissue modules with precision and stoichiometric competence still unmatched by man-made devices. Such technologies have led to the development of cohesive cell-assembled prototypes, held together by cell-cell and cell-ECM junctions, for skin^7^,^8^,^9^, blood vessel^10^,^11^ and cornea^12^ that have demonstrated reparative/regenerative efficacy, even in clinical setting.

Over 30 years have passed since the development of the first tissue equivalent *in vitro*^7^,^8^,^9^; it is now evidenced that this scientific breakthrough was not followed by a similar magnitude technological advancement. Indeed, we have now recognised that the major culprit in clinical translation and commercialisation of cell-based therapies is the creation of functional *in vitro* microenvironments that, by imitating features of the tissue from which the cells were extracted from, will facilitate cell phenotype, function and therapeutic potential maintenance *ex vivo*^13^. Thus, bioinspired research efforts recruit pioneered technologies in biophysical cues (e.g. surface topography, substrate elasticity), biochemical beacons (e.g. oxygen tension, ascorbic acid supplementation) and biological signals (e.g. growth factor supplementation, co-culture systems) to maintain permanently differentiated cells phenotype and/or to accurately direct stem cells lineage commitment for the creation of functional tissue facsimiles *in vitro*^14^,^15^,^16^,^17^.

Despite the significant advancements that have been achieved to-date, the development of an implantable device remains notoriously difficult and prohibitively expensive, as markedly long *ex vivo* culture times are required (e.g. 196 days for blood vessel)^10^ that are associated with loss of native phenotype and cellular senescence^18^,^19^. Herein, we hypothesise that macromolecular crowding (MMC), a biophysical phenomenon known to accelerate biological processes by several orders of magnitude^20^,^21^,^22^, will enhance protein turnover and amplify the production of ECM-rich supramolecularly assembled tissue equivalents. Specifically, *in vivo*, cells inhabit in the dense intertwined network of the ECM, where the proteinase-specific conversion of procollagen to collagen type I is rapid^23^. In the dilute culture media, the conversion of water soluble procollagen to water insoluble collagen type I is very slow; thus prolonged culture times are required to create an implantable device (Fig. 1). Thus, the addition of inert macromolecules in the culture media, by imitating the dense extracellular space, will enable the accelerated production of ECM-rich living substitutes (Fig. 1). To validate our hypothesis, human corneal fibroblasts (HCFs) were used for very first time as exemplary and the influence of a Ficoll™ cocktail (FC) as a means of MMC on collagen type I, the most abundant ECM protein of corneal stromal, biosynthesis was assessed.

## Results

### Identification of optimal newborn calf serum (NBCS) concentration for maximum ECM deposition

Sodium dodecyl sulphate polyacrylamide gel electrophoresis (SDS-PAGE; Fig. S1) and complementary densitometric analysis (Fig. S2) demonstrated that at all NBCS concentrations collagen type I remained in the media in the absence of FC, whilst in the presence of FC, collagen type I was deposited in the cell layer. It was further observed that collagen type I content remained constant or reduced as a function of increased NBCS concentration at given time point in culture. This reduction in collagen type I content was attributed to the enhanced matrix metalloproteinase-2 (MMP-2) activity, as revealed by gelatin zymography (Fig. S3). The highest (*p* < 0.0001) collagen type I deposition was achieved after culturing HCFs for 6 days in the presence of FC. No significant difference (*p* > 0.05) in collagen type I deposition was observed among the different NBCS concentrations (0 to 10%) at any time point tested (2, 4 and 6 days). Phase contrast microscopy (Fig. S4) revealed that HCFs maintained their spindle-shaped morphology for all experimental conditions (e.g. presence/absence of FC; 0%–10% NBCS; 2–6 days in culture). Further, the addition of FC did not affect cell metabolic activity (Fig. S5A) and cell viability (Fig. S5B and Fig. S5C) independently of the experimental condition.

### Identification of optimal serum origin for maximum ECM deposition

**Figure 2:**
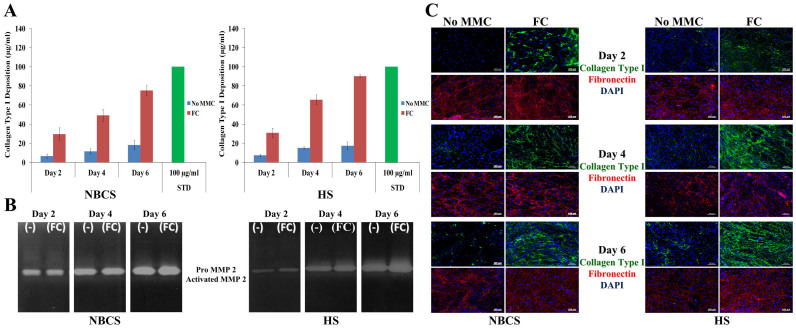
MMC accelerates ECM deposition in HCFs culture in the presence of NBCS and HS. (A) Densitometric analysis of SDS-PAGE confirmed the high collagen type I deposition in the cell layer as early as 2 days in culture. Collagen type I deposition was consistently increased up to 6 days in culture (longer time point assessed). (B) Gelatin zymography detected the 68–72 kDa proMMP-2 and the 62 kDa MMP-2 active form. Both forms of enzyme were higher in NBCS, which explains the enhanced ECM deposition in the presence of HS. (C) ICC analysis further collaborated the enhanced collagen type I deposition in the presence of FC, whilst fibronectin, the template for collagen deposition, was not affected.

To avoid xenogeneic contaminants, the influence of human serum (HS), as an alternative to NBCS, was assessed at 0.5%, in presence and absence of FC for 2, 4 and 6 days. SDS-PAGE (Fig. S6) and complementary densitometric analysis (Fig. 2A) showed that in the absence of FC collagen type I remained in the media and its content remained constant as a function of time in culture. This indifference in collagen type I content was attributed to the increased MMP-2 content, as revealed by gelatin zymography (Fig. 2B). In the presence of FC, collagen type I was deposited at the cell layer and its content was increased as a function of time in culture. The highest (*p* < 0.0001) collagen type I deposition was achieved after culturing HCFs for 6 days in the presence of FC. Significant increase in collagen type I (*p* < 0.001) deposition was observed in the presence of HS, as compared to NBCS at 4 and 6 days. The increase in collagen deposition in the presence of HS was attributed to the lower MMP-2 content of HS (Fig. S7).

Immunocytochemistry (ICC) analysis (Fig. 2C) further corroborated the high collagen type I deposition in the presence of FC and its relative increase as a function of time in culture, independently of the serum origin. No difference in fibronectin deposition was observed. Phase contrast microscopy (Fig. S8) revealed that HCFs maintained their spindle-shaped morphology for all experimental conditions (e.g. presence/absence of FC; 0.5% NBCS/HS; 2–6 days in culture). Further, the presence of FC did not affect cell metabolic activity (Fig. S9A), cell viability (Fig. S9B and Fig. S9C) and DNA content (Fig. S9D), at any given time point, independently of the serum origin.

### Production and characterisation of ECM-rich HCFs supramolecular assemblies

**Figure 3:**
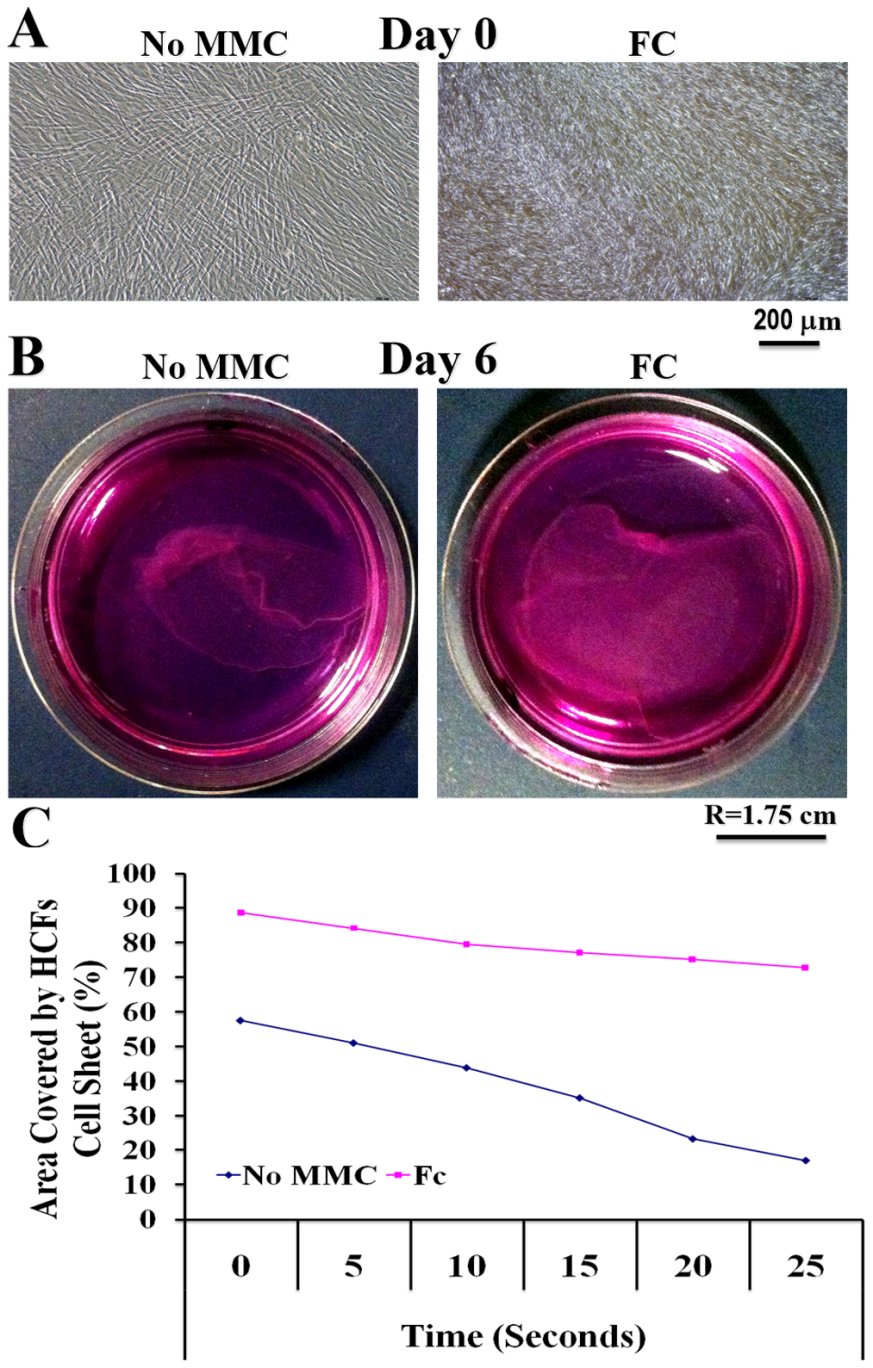
A poly(NIPAAM-co-NTBA) temperature-responsive copolymer allowed production and detachment of dense and cohesive corneal stromal layers. (A) The poly(NIPAAM-*co*-NTBA) temperature-responsive copolymer allowed attachment and spreading of HCFs under MMC and non-MMC conditions. (B) The poly(NIPAAM-*co*-NTBA) temperature-responsive copolymer allowed intact detachment of the *de novo* produced ECM-rich HCFs sheets after 6 days in culture. (C) The presence of FC, which resulted in enhanced ECM deposition, delayed the intact detachment of the ECM-rich HCFs sheets; nonetheless, complete detachment achieved within 45 min after temperature reduction.

Due to the abundant ECM deposition in the presence of FC, commercially available temperature-responsive N-isopropylacrylamide (NIPAAM) coating was not suitable for the detachment of intact ECM-rich HCFs supramolecular assemblies after 6 days in culture, although cell attachment and growth was not prohibited (Fig. S10). A 65% N-isopropylacrylamide/35% N-tert-butylacrylamide (NTBA) copolymer [poly(NIPAAM-*co*-NTBA)] allowed attachment (Fig. 3A) and detachment (Fig. 3B) of intact ECM-rich HCFs supramolecular assemblies after 6 days in culture in the presence of FC and 0.5% HS. Auxiliary time-lapse microscopy revealed that due to the abundance in deposited ECM under MMC conditions, the detachment rate of the ECM-rich HCFs supramolecular assemblies was slower than their non-MMC counterparts (Fig. 3C and S11).

**Figure 4:**
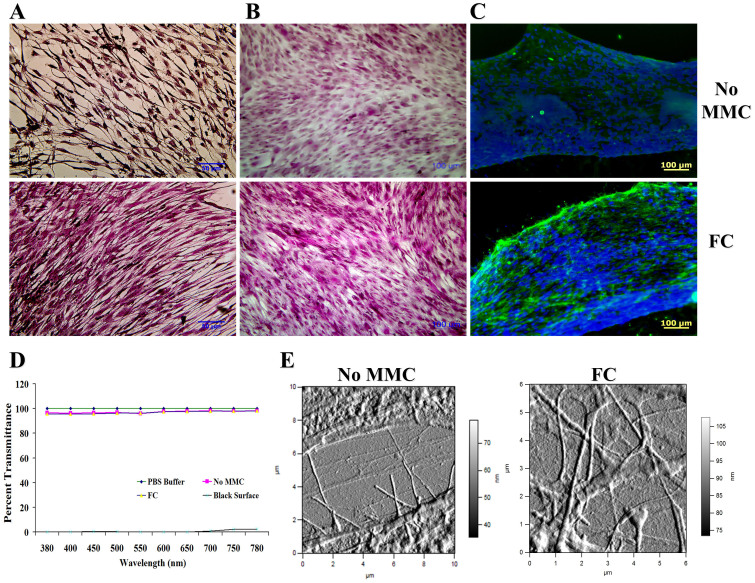
MMC allowed production of HCFs sheets with physiological, tissue-like characteristics. (A and B) Histological analysis using van Gieson's and Masson's trichrome staining demonstrate enhanced ECM deposition and tissue-like organization of the HCFs substitutes under MMC conditions. (C) ICC analysis for collagen type I further corroborates the enhanced collagen type I deposition under MMC conditions. (D) No significant difference in transparency between MMC HCFs sheets and PBS, control surface and non-MMC sheets was observed at 0.5% HS and after 6 days in culture. (E) AFM analysis at the intracellular regions (shown are amplitude images) further confirmed the high deposition of collagenous proteins exhibiting characteristic D-periodicity in the presence of FC.

Histological analysis using van Gieson's (Fig. 4A) and Masson's trichrome (Fig. 4B) staining and ICC analysis (Fig. 4C) further corroborated the enhanced collagen type I deposition under MMC conditions (0.5% HS, FC, 6 days in culture). Transmittance analysis (Fig. 4D) revealed no significant difference (*p* > 0.05) between the groups (non-MMC, 0.5% HS, 6 days in culture; MMC, 0.5% HS, 6 days in culture; PBS; control surface). Further atomic force microscopy analysis (Fig. 4E and S12) revealed the presence of abundant quartered staggered collagen type I fibrils at the intercellular regions of the MMC HCFs (0.5% HS, 6 days in culture).

**Figure 5:**
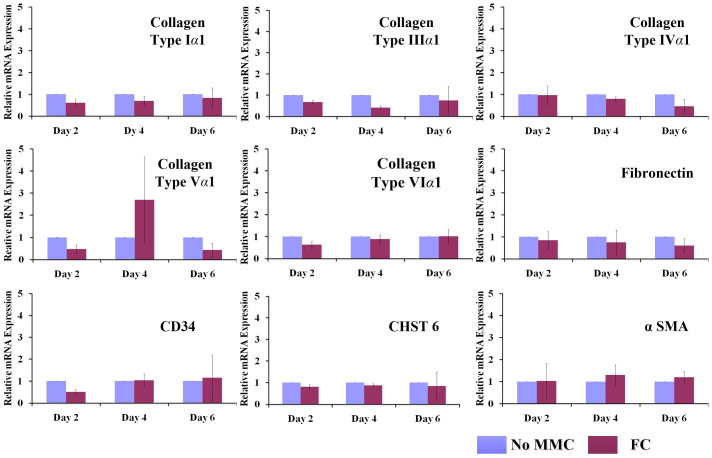
RT-qPCR analysis demonstrated that MMC did not affect gene expression profile of HCFs at respective time points. The expression of collagens (I, III, IV, V, VI); glycoproteins (fibronectin); phenotypic markers (CD34, CHST6); and unintended differentiation markers (*α* SMA) for all time points assessed (2, 4 and 6 days) remained unaffected under the conditions tested (0.5% HS, with or without FC).

**Figure 6:**
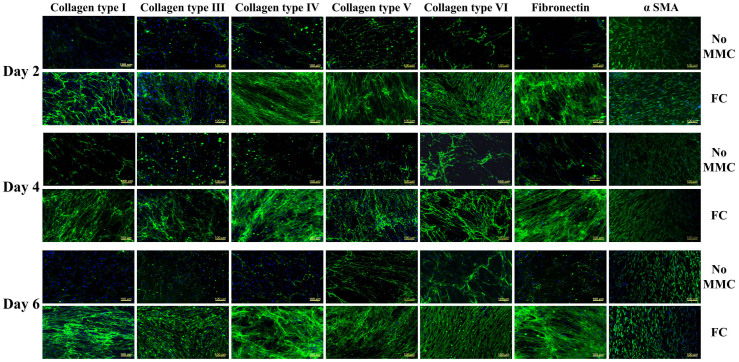
MMC enhanced ECM deposition in the cell layer of HCFs. Validation of proteomics analysis with ICC further confirmed the high deposition of collagenous proteins (I, III, IV, V, VI) and glycoproteins (fibronectin) in the presence of FC, whilst no difference was observed for unintended differentiation markers (*α* SMA) at all time points (2, 4 and 6 days). A high level of structural alignment is also evidenced.

MMC (0.5% HS, FC) did not affect (*p* > 0.05) gene expression analysis (Fig. 5) for collagens (I, III, IV, V, VI); fibronectin; phenotypic markers (CD34, CHST6); and unintended differentiation marker (*α* SMA) for all time points assessed (2, 4 and 6 days). Proteomics analysis revealed that under MMC conditions (0.5% HS, FC, 6 days), the total protein content in the cell layer was significantly increased (Table S2). Subsequent validation using ICC (Fig. 6) and complementary fluorescence intensity (Fig. S13) further confirmed the high deposition of collagenous proteins (I, III, IV, V, VI) and fibronectin under MMC conditions (0.5% HS, FC, 6 days in culture), whilst no difference was observed for unintended differentiation markers (*α* SMA) and phenotypic markers (CD34, keratocan; Fig. S14) for all time points (2, 4 and 6 days in culture).

### Low-density versus high-density culture

**Figure 7:**
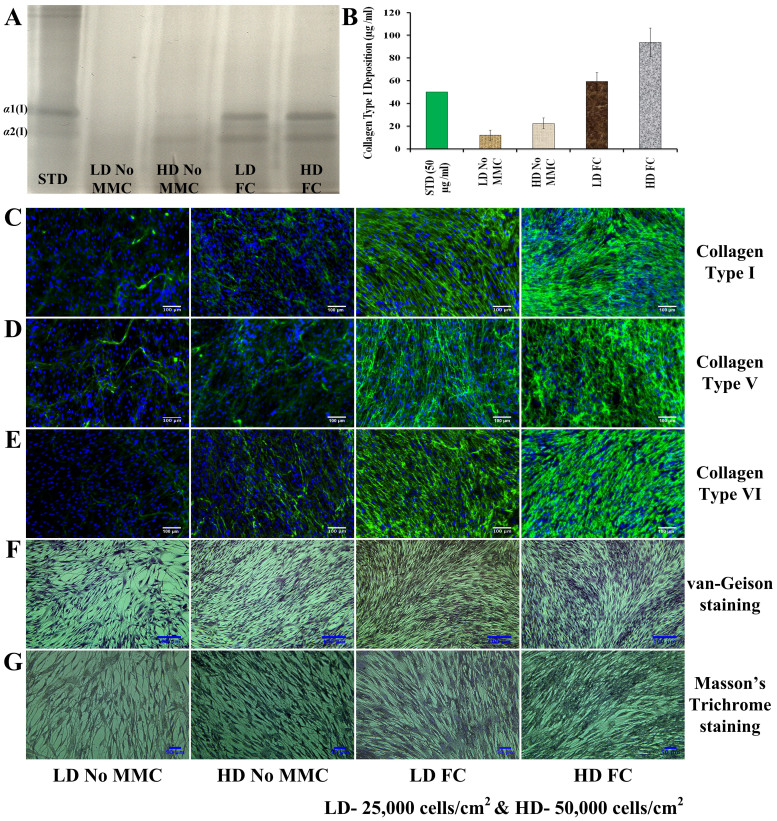
MMC is more effective in producing ECM-rich living substitutes than high-density non-crowded cultures. At day 6, MMC at low-density (LD; 25,000 cells/cm^2^) culture induced higher ECM deposition than non-crowded high-density (HD; 50,000 cells/cm^2^) culture and the highest ECM deposition was detected in the high-density culture in the presence of MMC, as evidenced by SDS-PAGE (A) and complementary densitometric (B) analysis, immunocytochemistry analysis (C, D, E) and staining assays (F, G).

At day 6, MMC at low-density (25,000 cells/cm^2^) culture induced higher ECM deposition than non-crowded high-density (50,000 cells/cm^2^) culture (Fig. 7). The highest ECM deposition was detected in the high-density culture in the presence of MMC (Fig. 7).

## Discussion

During development, cellular secretome creates a tissue-specific microenvironment that governs the various functions of the tissue. Cellular secretome also provides necessary signals for tissue repair and regeneration during or following injury. Therapeutic strategies based on the principles of tissue engineering by self-assembly aim to capture this specific reparative potential of cellular secretome. However, for such approaches to succeed, it is essential to create a functional *ex vivo* microenvironment that by emulating the tissue from which the cells were extracted from or are to be implanted in will maintain cell phenotype, function and therapeutic potential. Unfortunately, the still primitive culture conditions not only are associated with phenotypic drift, but also require a prolonged period of time for the development of an implantable device. Herein, we hypothesised that MMC, by imitating the dense extracellular space, will accelerate the deposition of tissue-specific ECM, whilst preserving cell phenotype. Given that tissue engineering by self-assembly strategies for corneal stromal require 4 to 8 weeks for the production of supramolecularly assembled monolayers^24^,^25^,^26^ and the readiness of corneal fibroblasts to trans-differentiate towards unintended myofibroblast lineage^27^,^28^,^29^,^30^,^31^,^32^, we used this cell type as model.

In the absence of MMC, collagen type I remained in the media, whilst in the presence of FC ample ECM deposition was evidenced in the cell layer as early as 48 h in culture, which persisted up to 6 days in culture (longer time point tested). This is in accordance to previous observations, where FC accelerated ECM deposition in human lung fibroblast culture^33^ and human bone marrow stem cell culture^34^. The efficacy of FC to induce accelerated ECM deposition lays on the fact that effectively excludes volume^35^,^36^, facilitating that way cleavage of *C*- and distant *N*-propeptides by their respective proteinases and therefore accelerated conversion of procollagen to collagen type I.

Tissue engineering by self-assembly supramolecular aggregates are customarily produced using copious amounts of animal sera (up to 30%). Our data indicate that excessive sera supplementation is counter-effective, due to the high amount of MMP-2 present that degrades deposited ECM. Given though that sera contain survival signals, mitogens and growth factors, all necessary for cell expansion, a balance needs to be achieved that will allow both cell survival and ECM deposition. Herein, we demonstrate that low serum concentration appears to achieve optimal balance between matrix deposition and cell growth, which is in accordance to previous results, where high serum supplementation resulted in phenotypic drift of corneal stromal cells^37^,^38^,^39^, tenocytes^40^ and retinal progenitor cells^41^, whilst low serum or serum-free media resulted in phenotype maintenance of dental-derived stem cells^42^, embryonic stem cells^43^ and mesenchymal stem cells^44^,^45^. Further, given the potential of interspecies transmission of disease and severe immune reactions associated with animal sera, our data further corroborate previous studies supporting the use of HS for clinically relevant cell therapies^46^,^47^,^48^,^49^,^50^.

The ample deposition of ECM in the presence of FC prohibited harvesting of intact supramolecularly assembled ECM-rich HCFs substitutes from the brush-like commercially available NIPAAM dishes. The efficiency of poly(NIPAAM-*co*-NTBA) copolymer to produce intact ECM-rich cell sheets is attributed to the decreased number of N – H residues, due to the additional steric hindrance induced by the addition of the NTBA group, which decreased hydrogen bonding and consequently decreased protein adsorption^51^, making cell detachment easier. Although the detachment rate of the MMC samples was slower than the non-MMC counterparts, a fully characterised poly(NIPAAM-*co*-NTBA) copolymer^52^,^53^,^54^ allowed harvesting of dense and cohesive tissue-like modules as a continuous sheet within 45 min by a simple switch of temperature from 37°C to 10°C. Subsequent morphological analysis revealed that these dense and cohesive supramolecularly assembled living substitutes produced within 6 days in culture in the presence of FC had intact cell-cell and cell-ECM junctions and achieved similar level of biomimicry as scaffold and scaffold-free substitutes that were produced after 3^55^, 4^24^, 9^56^ or even 11^57^ weeks in culture. Further, although traditional self-assembled living substitutes require prolonged culture time to form and mature, in the presence of macromolecular crowding, such processes are enhanced, as evidenced by the presence of *β*-bands on the gels.

Further gene and protein analysis assays demonstrated that FC did not affect expression levels of collagenous proteins, glycoproteins, phenotypic markers and unintended differentiation markers. It is worth pointing out that both MMC and non-MMC counterparts were negative for CD34 and keratocan at protein level and very low expression at gene level. It has been reported that the expression of these markers rapidly declines in low-density cultures^58^ after exposure to serum^37^,^38^,^39^,^59^ and as a function of time in culture^56^,^60^,^61^.

Here, we demonstrate that modulation of the *in vitro* microenvironment of HCFs with FC results in ECM-rich supramolecular assemblies within days rather than weeks or months in culture, without compromising fundamental cellular functions. This technology not only requires a lower cell number than multilayer cell sheets or high-density cultures, which is often not available, but also bypasses altogether such approaches that due to poor nutrient, oxygen and waste transport result in cell necrosis in the central cell-layers^62^. It further evades complex and expensive culture media that only temporarily will maintain phenotype, without actually increasing ECM deposition^63^,^64^,^65^,^66^,^67^.

## Methods

### Materials

Tissue culture consumables were purchased from Sarstedt (Ireland) and Nunc (Denmark). All other materials, including cell culture media, reagents and macromolecular crowders (Ficoll™ 70, Ficoll™ 400) were purchased from Sigma Aldrich (Ireland), unless otherwise stated. Live/Dead® viability/cytotoxicity kit and alamarBlue® cell metabolic activity kit were purchased from BioSource International, Invitrogen (Ireland).

### Corneal fibroblast culture

Primary HCFs (Innoprot, Spain) were cultured as per supplier protocol. Briefly 5,000 cells/cm^2^ were seeded on poly (L-lysine) coated tissue culture flasks maintained at 37°C with 5% CO_2_/95% air in a humidified incubator. Cells used in all experiments were between 3 and 5 passage.

### Macromolecular crowding treatment

HCFs were seeded in 24-well plates at 25,000 cells/cm^2^ density. When we compared low-density and high-density cultures, 25,000 cells/cm^2^ and 50,000 cells/cm^2^ were used, respectively. The next day, the media was replaced with fresh containing FC (Ficoll™ 70 + Ficoll™ 400: 37.5 mg/ml + 25 mg/ml) in the presence of 0.0 to 10.0% NBCS or HS. 100 *μ*M L ascorbic acid phosphate supplement was added in the media during the crowding experiment to enhance ECM synthesis. The influence of MMC was assessed at 2, 4 and 6 days in culture.

### Collagen deposition analysis

Media and cell layers were digested with porcine gastric mucosa pepsin (Sigma Aldrich, Ireland) for 2 h at 37°C with continuous shaking and subsequent neutralisation with 1 N NaOH. The samples for SDS-PAGE were prepared using appropriate dilution with distilled water and 5 × sample buffer. Finally, 15 *μ*l per sample solution per well was loaded on the gel (5% running gel/3% stacking gel) after 5 min heating at 95°C. Electrophoresis was performed in a Mini-PROTEAN Tetra Electrophoresis System (Bio-Rad, Ireland) by applying potential difference of 50 mV for the initial 30 min and then 120 mV for the remaining time (approximately 1 h). The gels were washed gently with ultra pure water and stained using silver stain kit (SilverQuest™, Invitrogen, Ireland) according to the manufacturer's protocol. Images of the gels were taken after brief washing with water. In order to quantify the cell-produced collagen type I deposition, the relative densities (GeneTools software, Syngene, Ireland) of collagen *α*1(I) and *α*2(I) chains were evaluated and compared to the *α*1(I) and *α*2(I) chain bands densities of standard collagen type I (Symatese Biomateriaux, France).

### Matrix metalloproteinase analysis

The presence of MMPs was evaluated using gelatin zymography. Briefly, at the end of each cell culture time point, media were collected and mixed with non-reducing SDS sample buffer (125 mM Tris-HCl, pH 6.8; 20% glycerol; 2% SDS; 0.002% bromophenol blue) and fractionated by SDS-PAGE using 10% gels containing 0.1% gelatin. After electrophoresis, the gels were washed with two incubations in 2.5% Triton X-100 for 30 min. The gels were incubated for 18 h at 37°C in a reaction buffer containing 50 mM Tris, pH 7.4; 5 mM CaCl_2_; 1 *μ*M ZnCl_2_ to promote recovery of protease activity were and then stained with 0.5% Coomassie G250 brilliant blue for 30 min. Images of the gels were taken after de-staining with 30% ethanol/10% acetic acid. These gelatin zymography gels bands were compared for relative expression of MMP-2. Uncultured media with various percentages of NBCS and HS were also used as controls in respective experiments.

### Cell morphology analysis

Cell morphology was assessed using phase contrast microscopy (Olympus IX81 inverted microscope, Japan).

### Cell metabolic activity and viability assessment

The influence of MMC on cell metabolic activity and viability was assessed using alamarBlue® and Live/Dead® assays, as per manufacturer's guidelines. Briefly, cell culture media containing 10% alamarBlue® reagent was added to various samples after removing the culture medium and brief washing with PBS and then incubated for 4 h to allow for the reduction of resazurin dye by active cells in cellular metabolism assay. Following incubation, 100 *μ*l of medium samples were transferred into a black 96-well plate. Fluorescence of the media was determined using a micro-plate reader (Varioskan Flash, Thermo Scientific, UK) at excitation and emission 570 nm/600 nm wavelength. The level of metabolic activity was calculated using % reduction of dye, according to the supplier's protocol and compared with the respective control samples. The cell viability was determined using Live/Dead® viability kit (Invitrogen, Ireland). The cells were incubated with calcium AM and ethidium homodimer solution (2 *μ*M calcein-AM and 4 *μ*M EthD-1) in PBS according to manufacturer's staining protocol for at least 30 min. The cell layers were washed in fresh PBS to remove excess dye. Following that, fluorescence images were taken using an Olympus IX81 inverted fluorescence microscope.

### DNA quantification

DNA quantification was carried out using Quant-iT™ PicoGreen® dSDNA assay kit (Invitrogen, Ireland) according to the manufacturer's protocol. Briefly, DNA was extracted using three freeze-thaw cycles after adding 200 *μ*l of nucleic acid free water per well (24 well plate). The cell suspension was subsequently transferred to cold eppendorf tubes and was centrifuged for 5 minutes at 12000 rpm. 25 *μ*l were then transferred into 96-well plate containing 75 *μ*l of 1 × TE buffer. A standard curve was generated using 0, 7.8, 15.6, 31.2, 62.5, 125, 250 and 500 *μ*g/mL DNA concentrations. 100 *μ*l of a 1:200 dilution of Quant-iT™ PicoGreen® reagent was added to each sample and the plate was read using a micro-plate reader (Varioskan Flash, Thermo Scientific, Ireland) with an excitation wavelength of 480 nm and an emission wavelength of 525 nm.

### Immunocytochemistry analysis

Cells were seeded in 4-well-chamber Lab-Tek™ II slide and MMC was carried out as described above. At the end of each culture time point, cells were fixed with 3% formaldehyde in PBS (pH 7.4). After fixation, the specimens were washed three times in PBS. The fixed cell layers were incubated with 3% BSA for 30 min to stop the non-specific binding of proteins. Cell layers were incubated with primary antibodies diluted in PBS for 90 min at room temperature. Rabbit collagen type I, III, IV, V and VI (Abcam, UK; 1:200) and mouse anti-fibronectin antibody (Sigma Aldrich, Ireland; F7387 1:200) were used to detect the expression of various collagens and fibronectin respectively. Mouse anti-actin, *α* smooth muscle antibody (1:400 dilution), mouse CD34 antibody (1:50 dilution) and mouse keratocan antibody (1:50 dilution) were used to detect *α* SMA, CD34 and keratocan expression in HCFs culture. Methanol fixing (cold methanol at −20°C for 5 min) was also used after fixation to detect CD34. After primary incubation, the samples were incubated with secondary antibody for 30 min at room temperature. The secondary antibodies used were Alexa Fluor® 488, chicken anti rabbit or donkey anti mouse respectively for rabbit and mouse antibodies at the 1:400 dilutions (Invitrogen, Ireland). Antibody incubation was followed by three washes in PBS. For nuclear staining, DAPI (4′,6-diamidino-2-phenylindole) was used at 1:4000 dilution (Invitrogen, Ireland). Finally, the cover slips were mounted on glass slides with VectaShield (Vector Laboratories, UK) for direct observation. The images were taken using an Olympus IX81 inverted fluorescence microscope using 10 × objective. The intensity of the fluorescence was evaluated using the Scope-Pro Plus software.

### Cell sheet production

Poly(NIPAAM-*co*-NTBA) was dissolved in anhydrous ethanol at 40 *μ*g/ml and subjected to continuous shaking overnight. This acrylamide solution was mixed with poly(L-lysine) (100 *μ*g/ml) in 1:1 V/V ratio and again allowed to shake overnight to mix properly. 100 *μ*l of the mixed solution was deposited onto each of the petri dishes, which were left in ethanol soaked desiccator overnight. The dishes were further dried in 600 mBar vacuum oven at 40°C for at least 4 h. Cell culture was carried out as described above after mild UV sterilisation of the petri dishes for 2 h. Following culture, a temperature-controlled plate was used to induce detachment and subsequent cell-sheet harvesting.

### Histological analysis using van Gieson staining

After 6 days in culture, samples were fixed with 3% paraformaldehyde for at least 30 min. The samples were then incubated with Weigert working solution for 10 min after 3 brief PBS washings. Finally, the samples were incubated in van Gieson's solution for 2–3 min, after brief PBS washing. The samples were dehydrated using 95% alcohol, absolute alcohol and xylene. Images were taken using the Olympus microscope (BX 51) at 100×.

### Histological analysis using Masson's Trichrome staining

Samples were fixed in Bouin's solution for 1 h at 56°C after fixation. The samples were then incubated in Weigert's iron haematoxylin staining for 10 min, followed by rinsing with running tap water. The samples were then washed with distilled water, after 10 min in warm running tap water. The samples were incubated in Biebrich scarlet-acid fuchsin solution for 10 to 15 min and washed again with distilled water. Phosphomolybdic-phosphotungstic acid solution was used for further 15 min and the samples were then transferred directly to aniline blue solution and stained for 10 min, rinsed briefly in distilled water and then in 1% acetic acid solution for 5 min. The samples were dehydrated quickly through 95% ethyl alcohol, absolute ethyl alcohol (these step wipes off Biebrich scarlet-acid fuchsin staining) and cleared in xylene after brief washing in distilled water. Images were then taken using the Olympus inverted microscope (BX 51) at × 100.

### Atomic force microscopy analysis

HCFs were seeded in 4-well-chamber Lab-Tek™ II slides and cell culture took place as described above. After six days in culture, cell layers were washed with HBSS and fixed at room temperature for 15 min. The cell layers were then washed three times with PBS and serially dehydrated with 30%, 50%, 70%, 90% and 100% ethanol. Atomic force microscopy (MFP-3D, Asylum Research, USA) analysis was then performed using rectangular Si cantilevers (SSS-NCH, Nanosensors, Switzerland), each having a nominal resonance frequency of 330 kHz and a spring constant of 42 N/m. Images were recorded using amplitude modulation mode in an ambient environment after drying the samples with nitrogen.

### Light transmission

Light transmission was assessed using a previously described method^26^. Briefly, the media was aspirated and 100 *μ*l PBS was added in each sample well after PBS washing. PBS alone was used as control blank. For zero absorbance and 100% absorbance, the wells without PBS or cells and black dye were taken as control; this black dye showed approximately 100% absorbance (0% transmittance), while the absorbance value in air was taken as 100% transmittance. The optical density of the samples was measured using a spectrophotometer (Varioskan Flash, Thermo Scientific, Ireland) at 380–780 nm wavelength with a resolution of 5 nm. The % transmission was calculated as: **% Transmittance = 100 (10ˆ-A)**, where A is absorbance measured.

### Gene expression (RT-qPCR) analysis

Total RNA was extracted using a modified Trizol isolation method at the given time points. Briefly, TriReagent® (Invitrogen, Ireland) was added to the cell layer after aspiration of medium from the culture and brief washing with PBS. The cellular layer was mechanically disrupted using gentle pipetting of tissue culture plate. Phase separation was conducted with chloroform and the total RNA contained in the aqueous phase was purified using RNeasy® mini kit column (QIAGEN, Hilden, Germany), according to the supplier's protocol. Three extractions were carried out for each sample and pooled at the end of the RNeasy protocol. The purity and quantity of total RNA were evaluated using an ultraviolet spectrometer (NanoDrop ND-1000 Spectrophotometer, Thermo Scientific, Ireland). Reverse transcription (RT) was performed using MJ Research PTC-200 DNA Engine system according to the manufacturer's protocol (Promega RT System, UK). The prepared cDNA was monitored using SYBRGreen master mix (QIAGEN, Hilden, Germany) by real-time PCR using StepOnePlus™ Real-Time PCR System (Applied Bioscience, Switzerland). Gene transcription was normalised to the transcription of housekeeping human 18S gene. The 2^−ΔΔCt^ method was used to calculate relative gene expression for each target gene at respective time point. Primers used for various collagens, fibronectin, CD34, CHST6 and *α* SMA are given in Table S1.

### Proteomics analysis

Total protein extraction from the cell layers was carried out using Qproteome™ mammalian protein preparation kit (Qiagen, UK). Briefly, cell layers were washed twice with PBS and scraped gently, using a cell scraper, in the presence of ice cold PBS and transferred to pre-chilled 1.5 ml tubes. This solution was centrifuged at 450 g for five min at 4°C. The cell layer was lysed gently with cell lysis buffer containing Benzonase® nuclease and protease inhibitor after discarding the supernatant. This suspension was centrifuged again at 14,000 g at 4°C for 10 min, after agitation in a rotary shaker for 5 min. After centrifugation, the supernatant was transferred into pre-chilled 0.5 ml tubes (Protein LoBind Tubes, UK) and freeze-dried. High-throughput proteomic profiling was performed using the 4-plex iTRAQ labelling kit, equipped with the 4700 MALDI TOF/TOF system (Applied Biosystems, USA). Briefly, the protein pellet from each sample was re-suspended in a protein dissolution buffer (10 mM triethylammonium bicarbonate, pH 8.5) and each protein sample was assigned to one of four isobaric tags as per the manufacturer's instructions. The tagged samples were pooled together and subjected to mass spectrometry analysis. Samples were fractionated using strong cation exchange and separated by reverse phase chromatography using the UltimatePlus NanoLC system (Dionex, USA). After reverse phase chromatography, the eluted protein fractions were spotted onto a MALDI plate (ABI 4800 OptiTOF, Applied Biosystems, USA). Using a Probot printing robot (Dionex, USA), the spotted plate was mixed with *α*-Cyano-4-hydroxycinnamic acid ionization matrix (Sigma Aldrich, Ireland) at a ratio of 1:2. Mass spectrometry analysis of the spotted plate was carried out an ABI 4800Plus MALDI-TOF/TOF tandem MS system (Applied Biosystems, USA). The time-of-flight of the protein was proportional to the molecular weight. The final read-out graph was a multi-peak spectrum with different peak intensities, which correspond to the relative protein amount. The peak location corresponds to the precise protein or peptide molecular weight. Data analysis was performed using Protein Pilot 2.0 software (Applied Biosystems, USA) on SWISS-PROT, TrEMBL (www.ebi.ac.uk/swissprot) and NCBI (www.ncbi.nlm.nih.gov/) non-redundant protein databases. The unused score was kept at more than 1.3 to get the confidence interval more than 90; relative peptide value at 95% was compared among samples. Selected ECM proteins detected in proteomics results were validated using ICC and fluorescent intensity, using Olympus IX81 inverted microscope.

### Statistical analysis

All results presented are mean ± SD. Statistical analysis (MINITAB™ version 16, Minitab, Inc., USA) was performed using two-sample t-test for pair wise comparisons or one away analysis of variance (ANOVA) for multiple comparisons after confirming: (a) the normal distribution from which each of the samples (Anderson-Darling normality test); and (b) the variances of the population of the samples were equal to one another (Bartlett's and Levene's tests for homogenicity of variance). Non-parametric statistics were used if above assumptions were violated and consequently Kruskal-Wallis test for multiple comparisons or Mann-Whitney test for 2-samples used. Each experiment was performed in biological triplicates in minimum three samples. Differences between selective experimental groups were considered statistically significant at *p* value < 0.05.

## Author Contributions

P.K. and D.Z. designed research; P.K., A.S., X.F. and E.C. performed experimental work; P.K., A.S., X.F., A.G. and Y.R. developed temperature responsive polymers and cell sheets; P.K., A.S., S.D. and D.Z. designed, performed, analysed and validated the proteomics study; P.K., A.S. and B.J.R. designed, performed and analysed AFM study; P.K., A.S. and D.Z. analysed data; A.P., M.R., L.J. and D.Z. discussed results; P.K. and D.Z. wrote the paper.

## Supplementary Material

Supplementary Information Supplementary Information

## Figures and Tables

**Figure 1:**
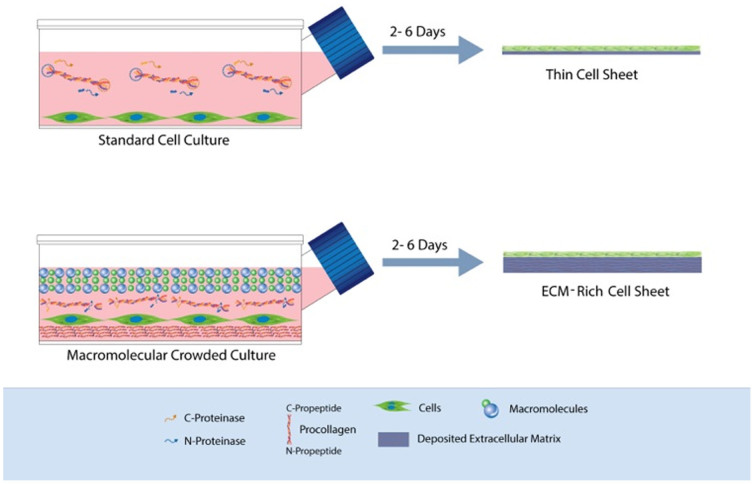
Schematic representation of how MMC enhances ECM deposition *in vitro* and its application in the development of ECM-rich cell sheet. Under standard cell culture conditions, the conversion of the water-soluble procollagen to insoluble collagen is very slow as the proteinases are deactivated before they cleave the specific *N*- and *C*- propeptides and/or the procollagen is dissolved before its conversion to collagen. After substantially long culture time, the cells will self-crowd the media and collagen is deposited in the cell layer. The addition of inert polydispersed macromolecules (presented as spheres with different diameters) in the culture media results in most effective volume occupancy; in increased relative density of procollagen and proteinases; in cleavage of the propeptides by their respectful proteinases; and finally in accelerated procollagen conversion to collagen and deposition of the former.
