# Supplementary material for: Macromolecularly crowded in vitro microenvironments accelerate the production of extracellular matrix-rich supramolecular assemblies
Source: Sci Rep. 2015 Mar 4;5:8729. doi: 10.1038/srep08729 (PMC4348624; doi:10.1038/srep08729)

**Supplementary Information**

Title

Macromolecularly crowded *in vitro* microenvironments accelerate the production of extracellular matrix-rich supramolecular assemblies

**Authors**

Pramod Kumar ^a^, Abhigyan Satyam ^a^, Xingliang Fan ^a^, Estelle Collin ^a^, Yury Rochev ^a^, Brian J. Rodriguez ^b^, Alexander Gorelov ^c^, Simon Dillon ^d^, Lokesh Joshi ^e^, Michael Raghunath ^f^, Abhay Pandit ^a^ Dimitrios I. Zeugolis ^a^*

**Affiliations**

^a^ Network of Excellence for Functional Biomaterials (NFB), National University of Ireland Galway (NUI Galway), Bioscience Research Building, Galway, Ireland

^b^ Conway Institute of Biomolecular & Biomedical Research, University College Dublin, Dublin, Ireland

^c^ School of Chemistry & Chemical Biology, University College Dublin, Dublin, Ireland

^d^ BIDMC Genomics, Proteomics, Bioinformatics and Systems Biology Center, Beth Israel Deaconess Medical Center, Harvard Medical School, Boston, USA

^e^ Alimentary Glycoscience Research Cluster, NUI Galway, Galway, Ireland

^f^ Department of Bioengineering, Faculty of Engineering, National University of Singapore Tissue Engineering Programme, Department of Biochemistry, Yong Loo Lin School of Medicine, National University of Singapore, Singapore

***Correspondence Author:** Dr Dimitrios Zeugolis, NFB, NUI Galway, Galway, Ireland

E-mail: dimitrios.zeugolis@nuigalway.ie; Office: +353-(0)-9149-3166; Fax: +353-(0)-9156-3991

**Table S1.** List of primers used for the RT-qPCR of various ECM molecules and HCF markers

| **Target Gene** | **Forward Primer** | **Reverse Primer** |
| --- | --- | --- |
| Collagen Type I*α*1 | CTGTAAACTCCCTCCATCCC | GTCCATGTGAAATTGTCTCCC |
| Collagen Type III*α*1 | CTGGGGAATGGAGCAAAAC | AAAGCAAACAGGGCCAAC |
| Collagen Type IV*α*1 | ACGACATCATCAAAGGGGAG | ACCCACCAATCCTGTAACAC |
| Collagen Type V*α*1 | ACCACCAAATTCCTCGACC | CCTCAAACACCTCCTCATCC |
| Collagen Type VI*α*1 | ATCGGACCTAAAGGCTACC | TTCTCCCCTTTCACCCATC |
| Fibronectin 1(FN1) | GGACCAGGACCAACAAAAAC | AGACACTAACCACATACTCCAC |
| Actin α2 Smooth Muscle, aorta (ACTA2)- Transcript Variant 1 | GCTGTTTTCCCATCCATTGTG | CCTCTTTTGCTCTGTGCTTC |
| Cluster Differentiation molecules 34 (CD34)- Transcript Variant 1 | ACATCTCTTACGCCCAACC | CACGTTTACCCAAAGAAGACC |
| N-acetylglucosamine-6-O-Sulfotransferase-6 (CHST6) | TTAGGCAGAAAAGGGGAGGG | TCAGGTGGAGGAAAGCAGAG |

**Table S2.** Mass spectrometry results of up-regulated (↑) and down-regulated (↓) proteins in HCFs culture in the presence of 0.5 % HS under non-MMC (control) and MMC conditions (FC) after 6 days in culture. PC indicates Peptide Count; ND indicates Not Detected.

**Table S2A.** Collagen and collagen related proteins in cell layer

| **Proteins** | **Accession** | **Location** | **Functions** | **95 % PC** | | **↓↑ Change** |
| --- | --- | --- | --- | --- | --- | --- |
|  |  |  |  | **Control** | **FC** |  |
| Collagen *α*1(I) chain OS=Homo sapiens GN=COL1A1 PE=1 SV=5 | sp|P02452|CO1A1_HUMAN | Extracellular matrix | Fibril forming collagen | ND | 62 | ↑ |
| Uncharacterized protein OS=Homo sapiens GN=COL6A3 PE=4 SV=1 | tr|E7ENL6|E7ENL6_HUMAN | Extracellular matrix | Fibril forming collagen | ND | 41 | ↑ |
| Collagen *α*2(I) chain OS=Homo sapiens GN=COL1A2 PE=1 SV=7 | sp|P08123|CO1A2_HUMAN | Extracellular matrix | Fibril forming collagen | ND | 24 | ↑ |
| Collagen *α*1(VI) chain OS=Homo sapiens GN=COL6A1 PE=1 SV=3 | sp|P12109|CO6A1_HUMAN | Extracellular matrix | Fibril forming collagen | ND | 13 | ↑ |
| Collagen *α*2(VI) chain OS=Homo sapiens GN=COL6A2 PE=1 SV=4 | sp|P12110|CO6A2_HUMAN | Extracellular matrix | Fibril forming collagen | ND | 5 | ↑ |
| Uncharacterized protein OS=Homo sapiens GN=COL12A1 PE=4 SV=1 | tr|D6RGG3|D6RGG3_HUMAN | Extracellular matrix | Fibril forming collagen | ND | 3 | ↑ |
| Collagen triple helix repeat-containing protein 1 OS=Homo sapiens GN=CTHRC1 PE=1 SV=1 | sp|Q96CG8|CTHR1_HUMAN | Extracellular matrix | May act as a negative regulator of collagen deposition | ND | 1 | ↑ |
| Collagen *α*1(V) chain OS=Homo sapiens GN=COL5A1 PE=1 SV=3 | sp|P20908|CO5A1_HUMAN | Extracellular matrix | Fibril forming collagen | ND | 1 | ↑ |

**Table S2B.** Fibronectin in cell layer

| **Proteins** | **Accession** | **Location** | **Functions** | **95 % PC** | | **↓↑ Change** |
| --- | --- | --- | --- | --- | --- | --- |
|  |  |  |  | **Control** | **FC** |  |
| Uncharacterized protein OS=Homo sapiens GN=FN1 PE=4 SV=1 | tr|E9PE77|E9PE77_HUMAN | Extracellular matrix | Fibril forming collagen | ND | 22 | ↑ |
| Uncharacterized protein OS=Homo sapiens GN=FN1 PE=4 SV=1 | tr|F8W7G7|F8W7G7_HUMAN | Extracellular matrix | Fibril forming collagen | 6 | ND | ↓ |

**Table S2C.** PGs in cell layer

| **Proteins** | **Accession** | **Location** | **Functions** | **95 % PC** | | **↓↑ Change** |
| --- | --- | --- | --- | --- | --- | --- |
|  |  |  |  | **Control** | **FC** |  |
| Basement membrane-specific heparan sulphate proteoglycan core protein OS=Homo sapiens GN=HSPG2 PE=1 SV=4 | sp|P98160|PGBM_HUMAN | Basement membrane | Adhesion promoter | ND | 1 | ↑ |

**Table S2D.** Laminin in cell layer

| **Proteins** | **Accession** | **Location** | **Functions** | **95 % PC** | | **↓↑ Change** |
| --- | --- | --- | --- | --- | --- | --- |
|  |  |  |  | **Control** | **FC** |  |
| Lamin A/C OS=Homo sapiens GN=LMNA PE=2 SV=1 | tr|Q6UYC3|Q6UYC3_HUMAN | Nucleus | Nuclear assembly, chromatin organisation, nuclear membrane and telomere dynamics | ND | 3 | ↑ |
| Filamin A, alpha (Actin binding protein 280) OS=Homo sapiens GN=FLNA PE=2 SV=1 | tr|Q5HY54|Q5HY54_HUMAN | Cytoplasm | Act as scaffold for a wide range of cytoplasmic signalling protein | ND | 2 | ↑ |

**Table S2E.** Tubulins in cell layer

| **Proteins** | **Accession** | **Location** | **Functions** | **95% PC** | | **↓↑ Change** |
| --- | --- | --- | --- | --- | --- | --- |
|  |  |  |  | **Control** | **FC** |  |
| Tubulin beta-2B chain OS=Homo sapiens GN=TUBB2B PE=1 SV=1 | sp|Q9BVA1|TBB2B_HUMAN | Cytoskeleton | Microtubule assembly | ND | 2 | ↑ |
| Tubulin, alpha 3, isoform CRA_c OS=Homo sapiens GN=TUBA1A PE=3 SV=1 | tr|G3V1U9|G3V1U9_HUMAN | Cytoskeleton | Microtubule assembly | 2 | 1 | ↓ |

**Table S2F.** Proteolytic activity in cell layer

| **Proteins** | **Accession** | **Location** | **Functions** | **95% PC** | | **↓↑ Change** |
| --- | --- | --- | --- | --- | --- | --- |
|  |  |  |  | **Control** | **FC** |  |
| Matrix metalloproteinase-14 OS=Homo sapiens GN=MMP14 PE=1 SV=3 | sp|P50281|MMP14_HUMAN | Membrane / Cytoplasm | May be involved in actin cytoskeleton reorganisation by cleaving PTK7 | ND | 2 | ↑ |
| Plasminogen OS=Homo sapiens GN=PLG PE=1 SV=2 | sp|P00747|PLMN_HUMAN | Cell membrane | Plasmin dissolves the fibrin of blood clots and acts as a proteolytic factor in a variety of processes: embryonic development, tissue remodelling | ND | 1 | ↑ |
| Alpha-2-macroglobulin OS=Homo sapiens GN=A2M PE=1 SV=3 | sp|P01023|A2MG_HUMAN | Secreted | Inhibit proteinase activity | ND | 4 | ↑ |

**Table S2G.** Other ECM and cytoskeleton proteins

| **Proteins** | **Accession** | **Location** | **Functions** | **95 % PC** | | **↓↑ Change** |
| --- | --- | --- | --- | --- | --- | --- |
|  |  |  |  | **Control** | **FC** |  |
| Keratin, type II cytoskeletal 1 OS=Homo sapiens GN=KRT1 PE=1 SV=6 | sp|P04264|K2C1_HUMAN | Cell membrane | Regulate the activity of kinases such as PKC and SR | ND | 2 | ↑ |
| Cytoskeleton-associated protein 4 OS=Homo sapiens GN=CKAP4 PE=1 SV=2 | sp|Q07065|CKAP4_HUMAN | ER/cell membrane | Mediates the anchoring of the endoplasmic reticulum to microtubules | ND | 1 | ↑ |
| Actin-related protein 3 OS=Homo sapiens GN=ACTR3 PE=1 SV=3 | sp|P61158|ARP3_HUMAN | Cytoskeleton | Regulation of actin polymerisation, formation of branched actin networks | ND | 1 | ↑ |
| Myosin, light polypeptide 6, alkali, smooth muscle and non-muscle, isoform CRA_d OS=Homo sapiens GN=MYL6 PE=4 SV=1 | tr|G3V1Y7|G3V1Y7_HUMAN | Cytoskeleton | Muscle contraction, protein binding, actin dependent ATPase activity and motor activity | ND | 1 | ↑ |
| Myosin-9 OS=Homo sapiens GN=MYH9 PE=1 SV=4 | sp|P35579|MYH9_HUMAN | Cytoskeleton | Cytokinesis, cell shape, and specialized functions such as secretion and capping | ND | 1 | ↑ |
| Lysosome-associated membrane glycoprotein 1 OS=Homo sapiens GN=LAMP1 PE=1 SV=3 | sp|P11279|LAMP1_HUMAN | Cell membrane | Carbohydrate metabolism | ND | 1 | ↑ |
| 60 kDa heat shock protein, mitochondrial OS=Homo sapiens GN=HSPD1 PE=1 SV=2 | sp|P10809|CH60_HUMAN | Mitochondrion matrix | Proteins folding | ND | 1 | ↑ |
| Trans-membrane protein 43 OS=Homo sapiens GN=TMEM43 PE=1 SV=1 | sp|Q9BTV4|TMM43_HUMAN | Endoplasmic reticulum | Maintaining nuclear envelope structure by organising protein complexes | ND | 1 | ↑ |
| Vimentin OS=Homo sapiens GN=VIM PE=1 SV=4 | sp|P08670|VIME_HUMAN | Cytoplasm | Involved with LARP6 in the stabilisation of type I collagen mRNAs for CO1A1 and CO1A2 | 8 | 17 | ↑ |
| Membrane-associated progesterone receptor component 2 OS=Homo sapiens GN=PGRMC2 PE=1 SV=1 | sp|O15173|PGRC2_HUMAN | Membrane | Steroidal receptor | ND | 1 | ↑ |
| Annexin A2 OS=Homo sapiens GN=ANXA2 PE=1 SV=2 | sp|P07355|ANXA2_HUMAN | Basement membrane/ECM | Involved in heat-stress response | 3 | 7 | ↑ |

**Table S2H.** Various enzymes

| **Proteins** | **Accession** | **Location** | **Functions** | **95 % PC** | | **↓↑ Change** |
| --- | --- | --- | --- | --- | --- | --- |
|  |  |  |  | **Control** | **FC** |  |
| Cytochrome c oxidase subunit 2 OS=Homo sapiens GN=MT-CO2 PE=1 SV=1 | sp|P00403|COX2_HUMAN | Inner membrane of mitochondria | Component of respiratory chain | ND | 1 | ↑ |
| Aminopeptidase N OS=Homo sapiens GN=ANPEP PE=1 SV=4 | sp|P15144|AMPN_HUMAN | Cell membrane | Final digestion of peptides generated from protein hydrolysis | ND | 1 | ↑ |
| Dolichyl-diphosphooligosaccharide--protein glycosyltransferase subunit 1 OS=Homo sapiens GN=RPN1 PE=1 SV=1 | sp|P04843|RPN1_HUMAN | Endoplasmic reticulum | Help in Protein modification; and protein glycosylation | ND | 1 | ↑ |
| Dihydropyrimidinase-related protein 2 OS=Homo sapiens GN=DPYSL2 PE=1 SV=1 | sp|Q16555|DPYL2_HUMAN | Cytoplasm | Neuronal development, axon growth/guidance, help in signalling by class 3 semaphorins, subsequent cytoskeleton remodelling | ND | 1 | ↑ |
| ATP synthase subunit beta OS=Homo sapiens GN=ATP5B PE=3 SV=1 | tr|F8VPV9|F8VPV9_HUMAN | Inner membrane of mitochondria | Catalytic activity in ATP production | ND | 1 | ↑ |
| Peptidyl-prolyl cis-trans isomerase B OS=Homo sapiens GN=PPIB PE=1 SV=2 | sp|P23284|PPIB_HUMAN | Endoplasmic reticulum | PPIases accelerate the folding of proteins. It catalyses the cis-trans isomerisation of proline imidic peptide bonds in oligopeptides. | ND | 1 | ↑ |
| ATP synthase subunit alpha, mitochondrial OS=Homo sapiens GN=ATP5A1 PE=1 SV=1 | sp|P25705|ATPA_HUMAN | Inner membrane of mitochondria | Catalytic activity in ATP production | ND | 2 | ↑ |

**Table S2I.** Miscellaneous proteins

| **Proteins** | **Accession** | **Location** | **Functions** | **95 % PC** | | **↓↑ Change** |
| --- | --- | --- | --- | --- | --- | --- |
|  |  |  |  | **Control** | **FC** |  |
| Histone H2A type 1-A OS=Homo sapiens GN=HIST1H2AA PE=1 SV=3 | sp|Q96QV6|H2A1A_HUMAN | Nucleus | Transcription regulation, DNA repair, DNA replication and chromosomal stability | 3 | 0 | ↓ |
| Peroxiredoxin-1 OS=Homo sapiens GN=PRDX1 PE=1 SV=1 | sp|Q06830|PRDX1_HUMAN | Cytoplasm | Redox regulation of the cell | 0 | 5 | ↑ |
| Histone H2B type F-S OS=Homo sapiens GN=H2BFS PE=1 SV=2 | sp|P57053|H2BFS_HUMAN | Nucleus | Transcription regulation, DNA repair, DNA replication and chromosomal stability | 0 | 5 | ↑ |
| Galectin-3 OS=Homo sapiens GN=LGALS3 PE=1 SV=5 | sp|P17931|LEG3_HUMAN | Cytoplasm/ Nucleus | Endothelial cells migration | 0 | 4 | ↑ |
|  |  |  |  |  |  |  |
| 78 kDa glucose-regulated protein OS=Homo sapiens GN=HSPA5 PE=1 SV=2 | sp|P11021|GRP78_HUMAN | Endoplasmic reticulum | Probably plays a role in facilitating the assembly of multimeric protein complexes inside the ER | 0 | 2 | ↑ |
| EMILIN-1 OS=Homo sapiens GN=EMILIN1 PE=1 SV=2 | sp|Q9Y6C2|EMIL1_HUMAN | Extracellular matrix | Anchoring smooth muscle cells to elastic fibres, Cell adhesive character | 0 | 1 | ↑ |
| Endoplasmin OS=Homo sapiens GN=HSP90B1 PE=1 SV=1 | sp|P14625|ENPL_HUMAN | Endoplasmic reticulum | Active role in the processing and transport of secreted proteins | 0 | 1 | ↑ |

**Fig. S1.** SDS-PAGE analysis revealed that in the absence of FC collagen remained in the media and its content was reduced as a function of time in culture and % NBCS. In the presence of FC, collagen I was deposited at the cell layer and its content was increased as a function of time in culture.

**
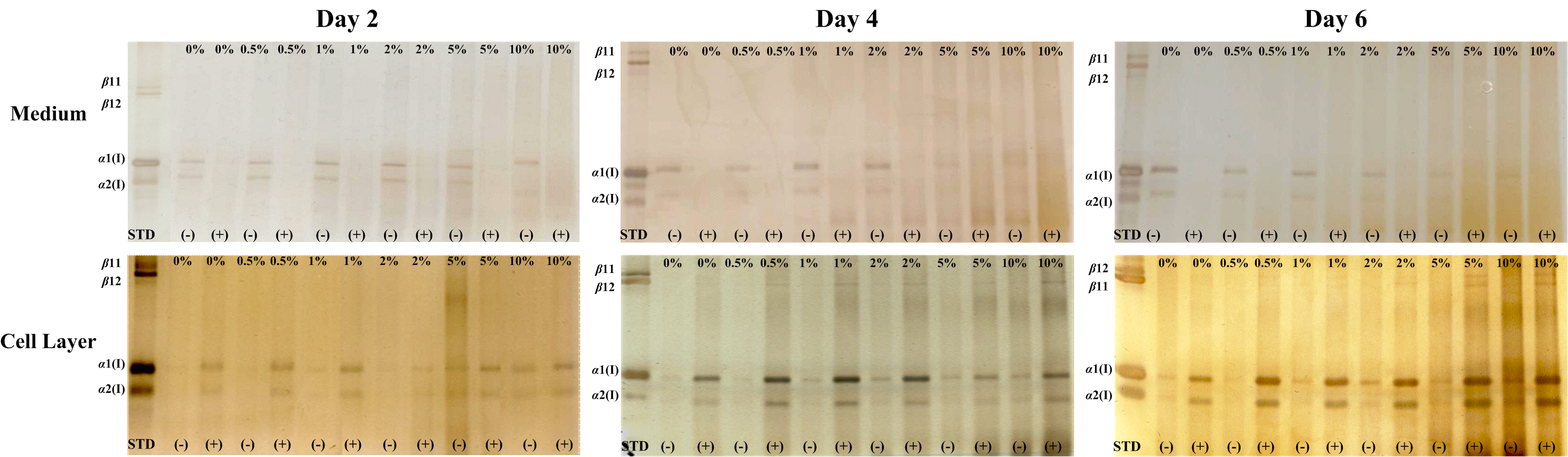
**

**Fig. S2.** Densitometric analysis of SDS-PAGE (**Fig. 1**) semi-quantitatively demonstrates the increased collagen deposition in the cell layer in the presence of FC as a function of time in culture. Increase NBCS concentration did not affect collagen deposition.


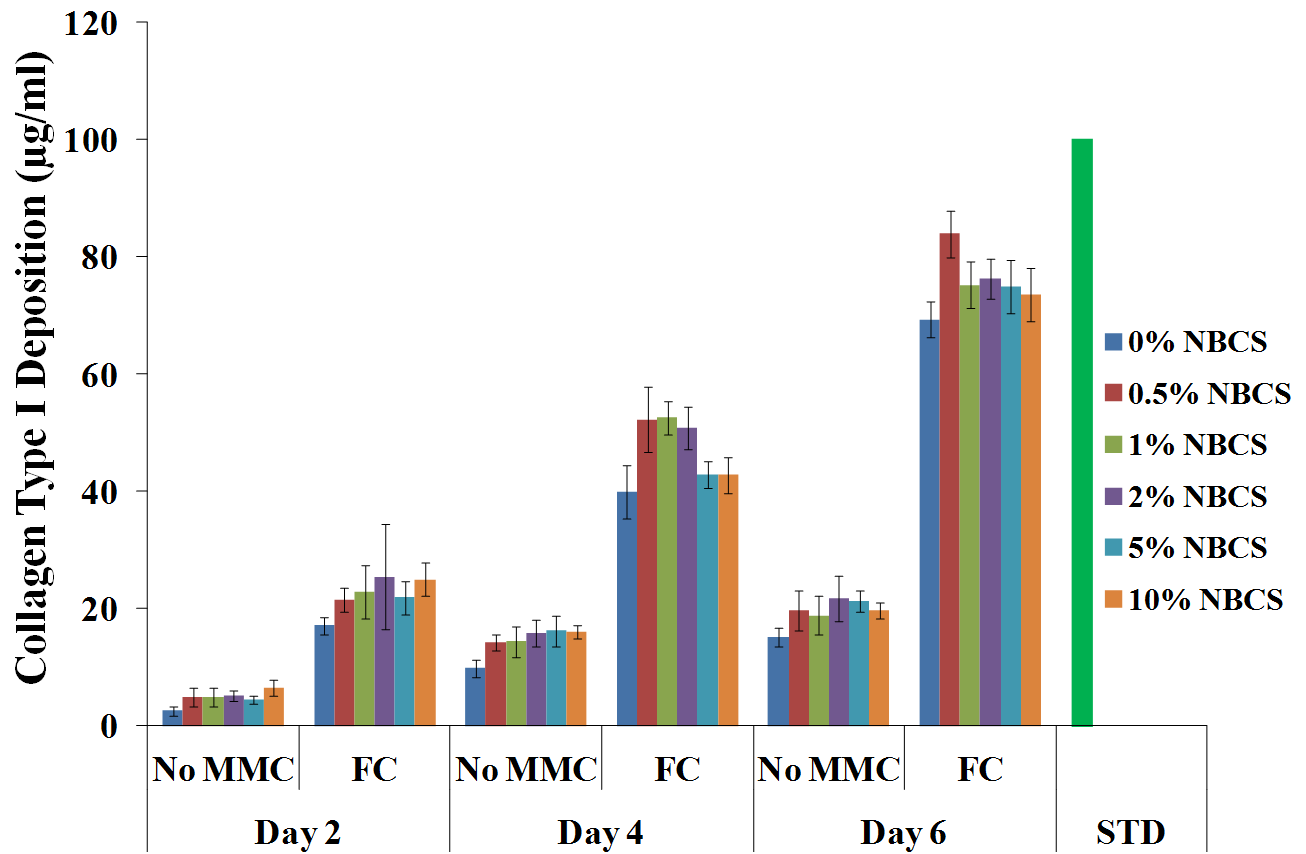


**Fig. S3.** The reduction in collagen content in the media as a function of increased NBCS concentration and time in culture is attributed to the enhanced MMP-2 content, as revealed by gelatin zymography.

**
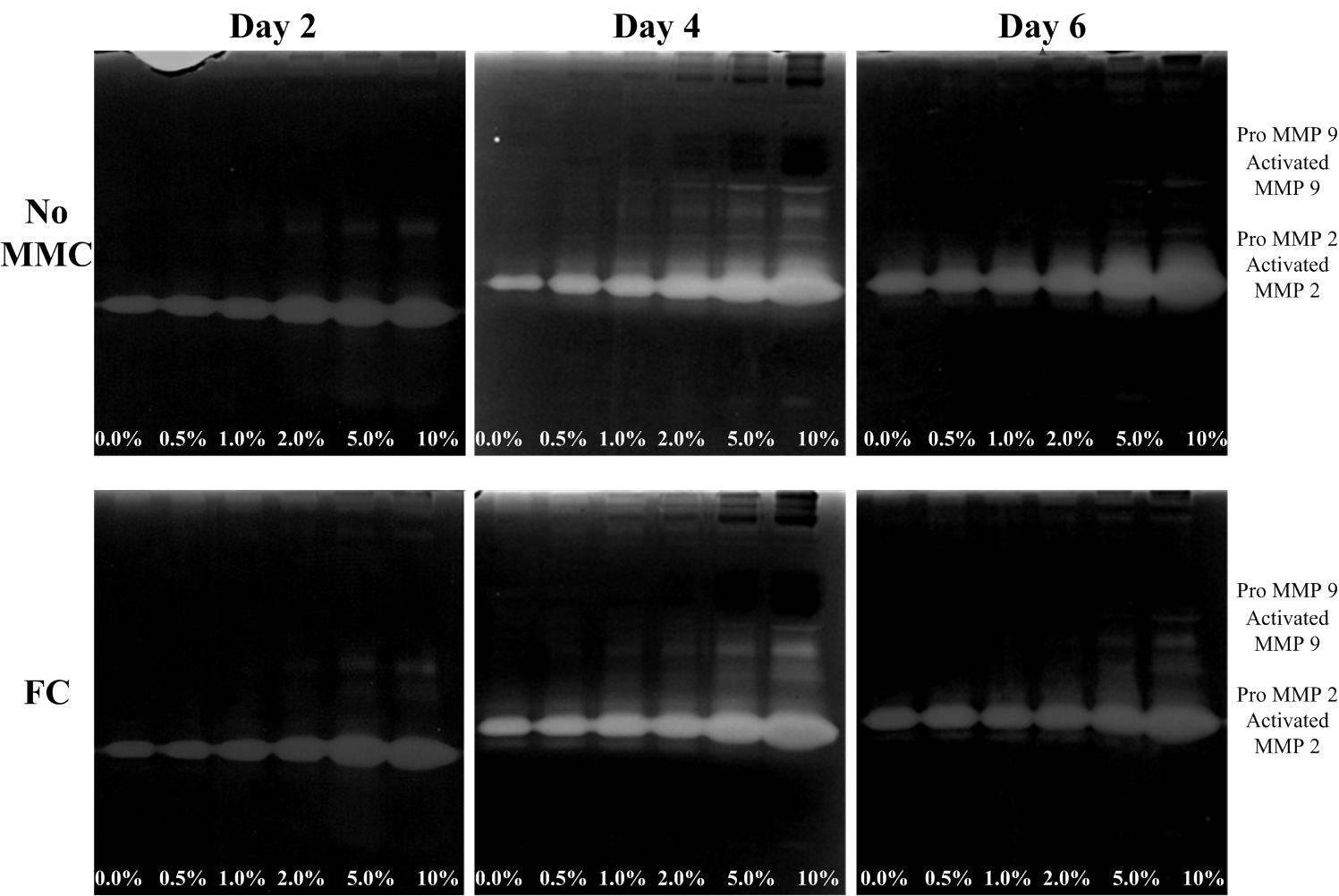
**

**Fig. S4.** Phase contrast microscopy revealed that HCFs maintained their spindle-shaped morphology for all time points (2, 4 and 6 days) independently of the presence or absence of FC and NBCS concentration (0 - 10 %).

**
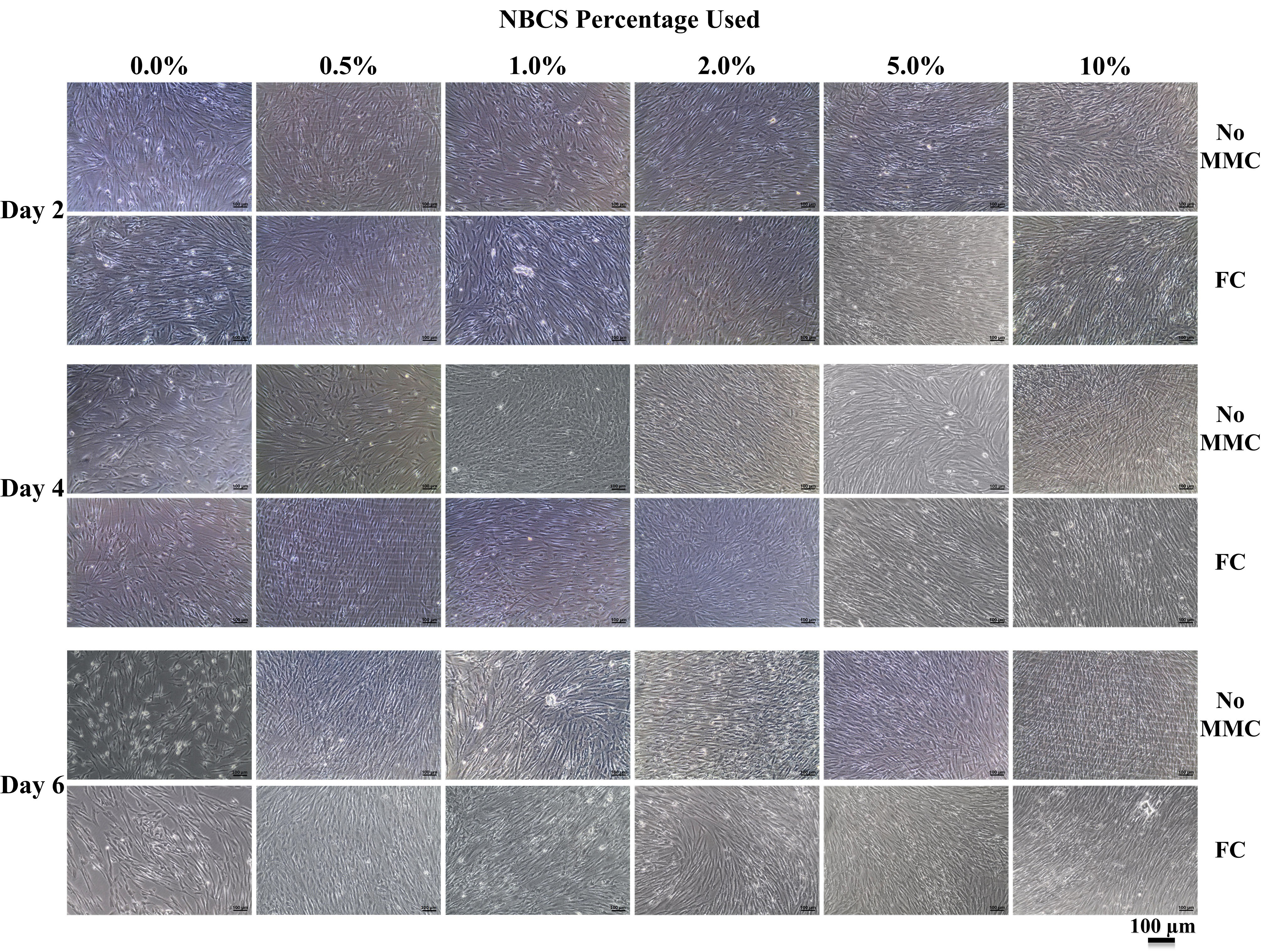
**

**Fig. S5.** HCF metabolic activity and viability as a function of FC presence and NBCS concentration. **(A)** MMC (FC) did not affect cellular metabolic activity, as revealed by alamarBlue® assay. **(B and C)** MMC (FC) did not affect cell viability, as revealed by Live/Dead® assay.

**
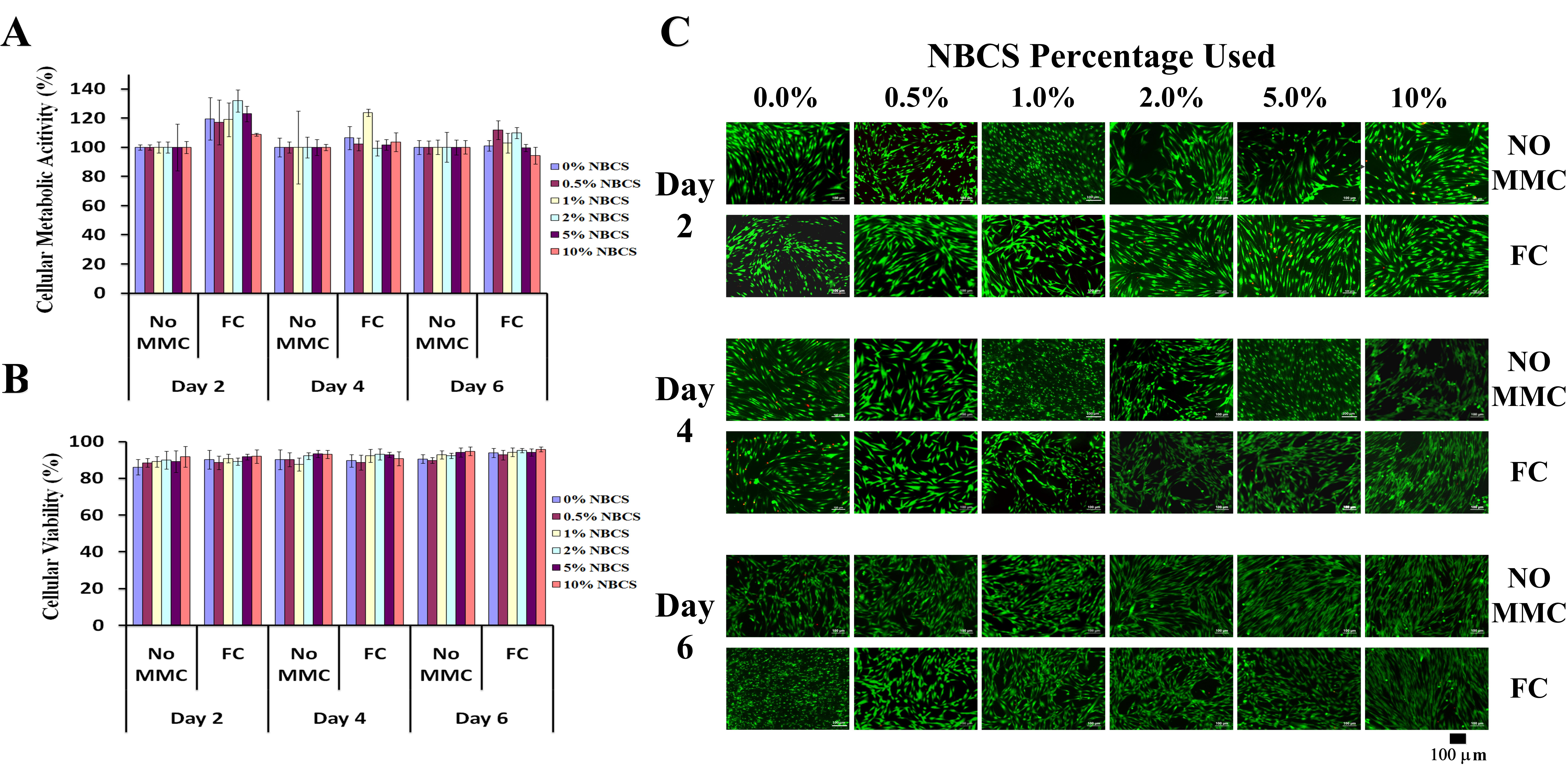
**

**Fig. S6.** To avoid xenogeneic contaminants, the influence of human serum (HS), as an alternative to NBCS, was assessed at 0.5 %, in presence and absence of FC for 2, 4 and 6 days. SDS-PAGE analysis showed that in the absence of FC collagen remains in the media and its content is reduced as a function of time in culture. In the presence of FC, collagen I was deposited at the cell layer and its content is increased as a function of time in culture.


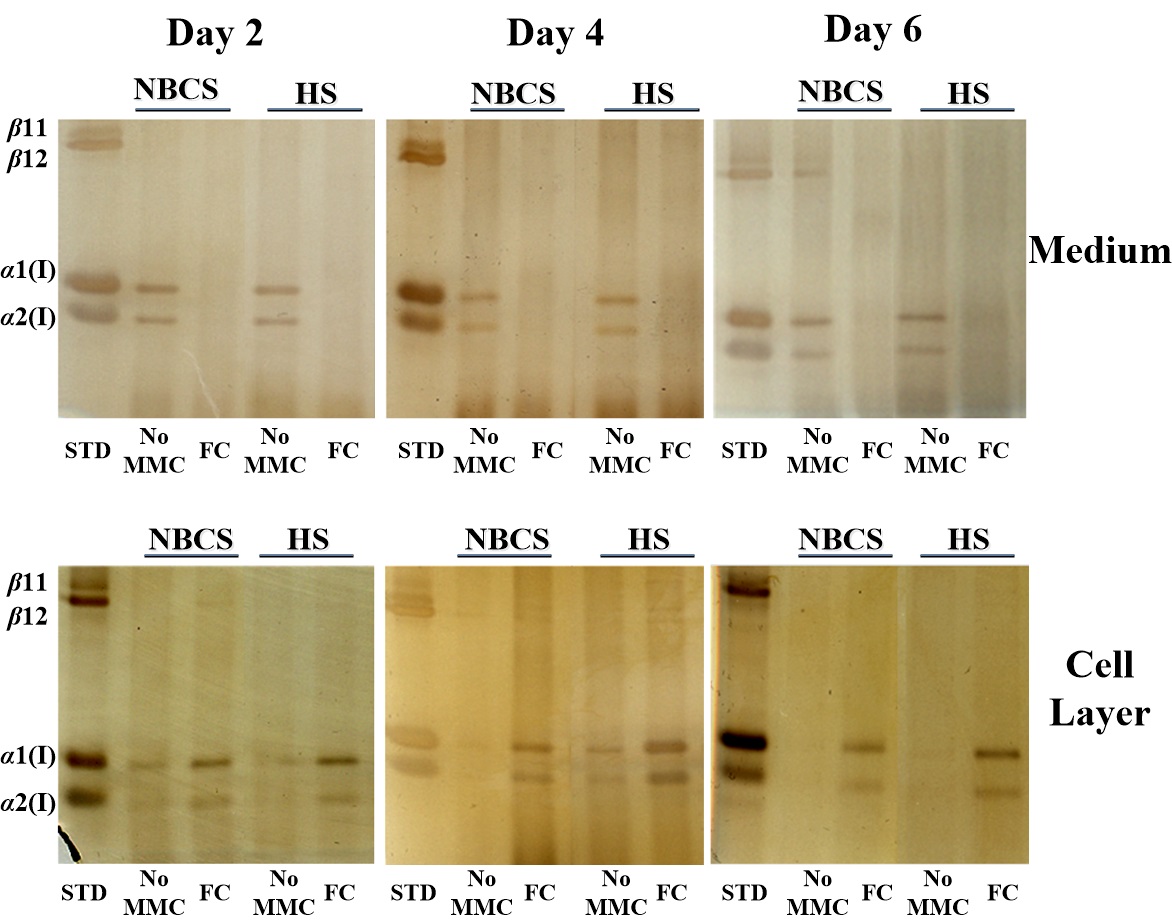


**Fig. S7.** Gelatin zymography of the medium containing various concentration of human serum or animal serum (0 - 10%) demonstrates that inherent high MMP activity of animal sera.

###
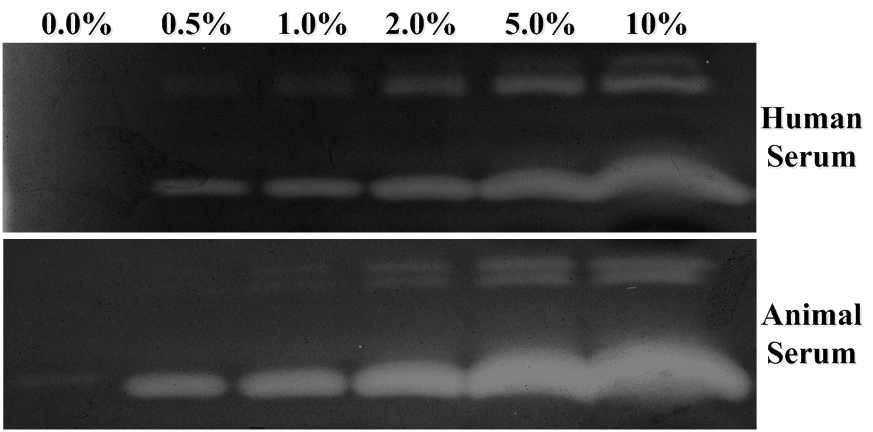


**Fig. S8.** Phase contrast microscopy revealed that HCFs maintained their spindle-shaped morphology for all time points (2, 4 and 6 days) independently of the presence or absence of FC, NBCS or HS.


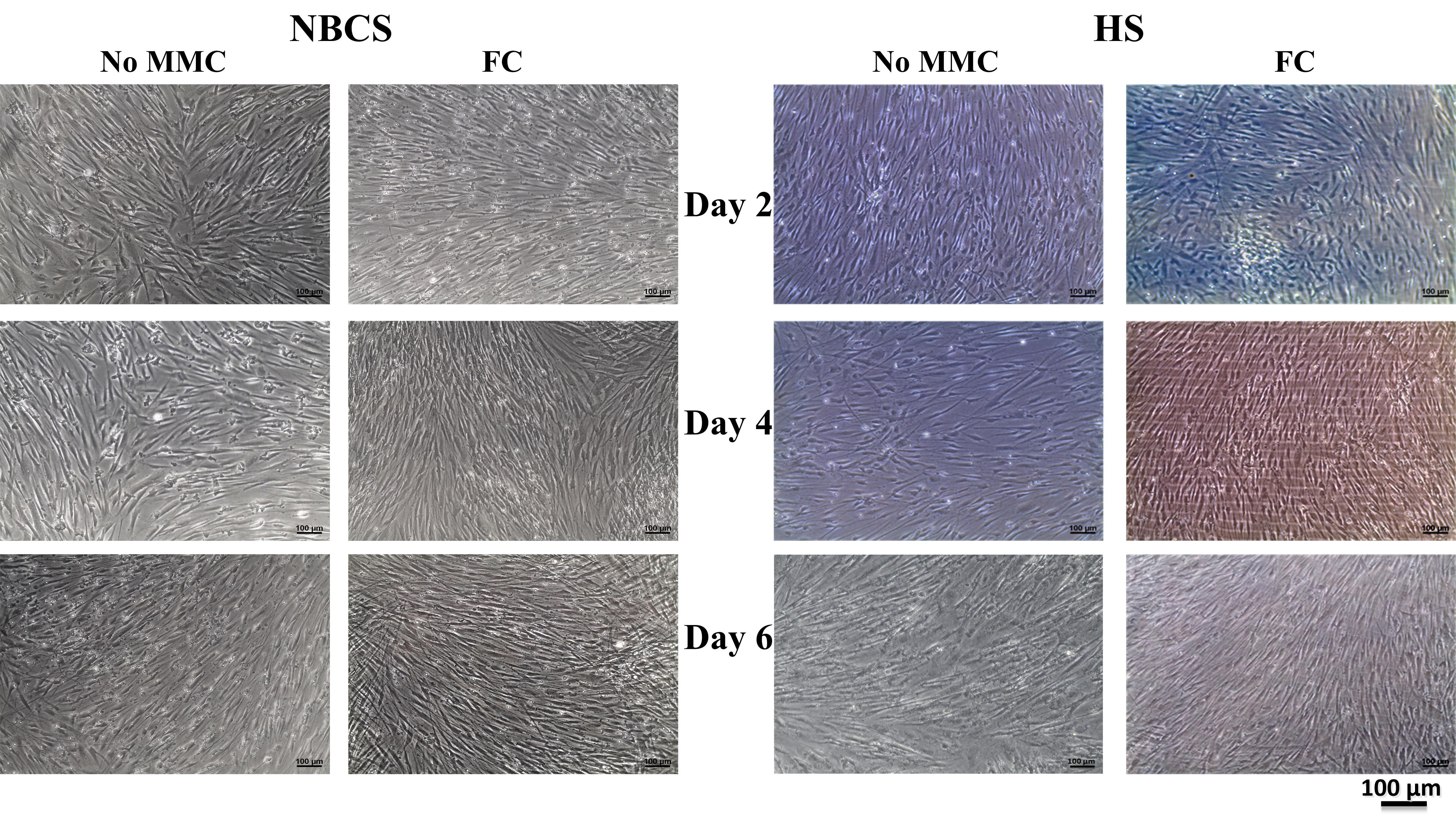


**Fig. S9.** HCF metabolic activity, viability and DNA assessment. **(A)** MMC (FC) did not affect cellular metabolic activity (A), viability (B, C) and DNA content (D) at a given time point, independently of the serum origin, as revealed by alamarBlue® assay, Live/Dead® assay and Quant-iT™ PicoGreen® dSDNA assay, respectively.





**Fig. S10.** Due to the abundance in ECM deposition in the presence of 0.5 % HS and FC, commercially available temperature-responsive NIPAAM-coated dishes, although allowed cell attachment and growth (**A** and **B**), did not allow detachment of intact ECM-rich HCFs after 6 days in culture (**C, D**).

**
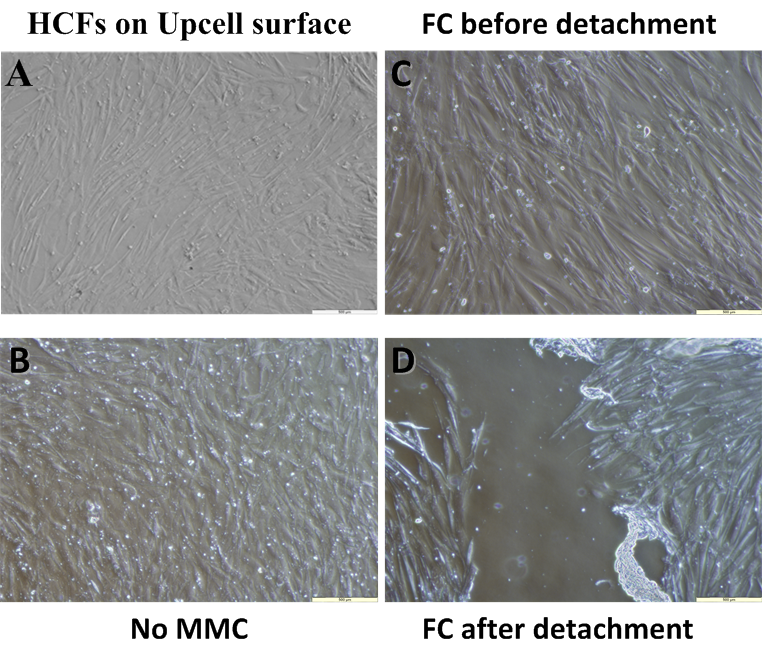
**

**Fig. S11.** Time-lapse microscopy demonstrated slow detachment of HCFs cell sheets treated with MMC (FC) due to the enhanced ECM deposition, after reduction of temperature below to lower critical solution temperature of copolymer.


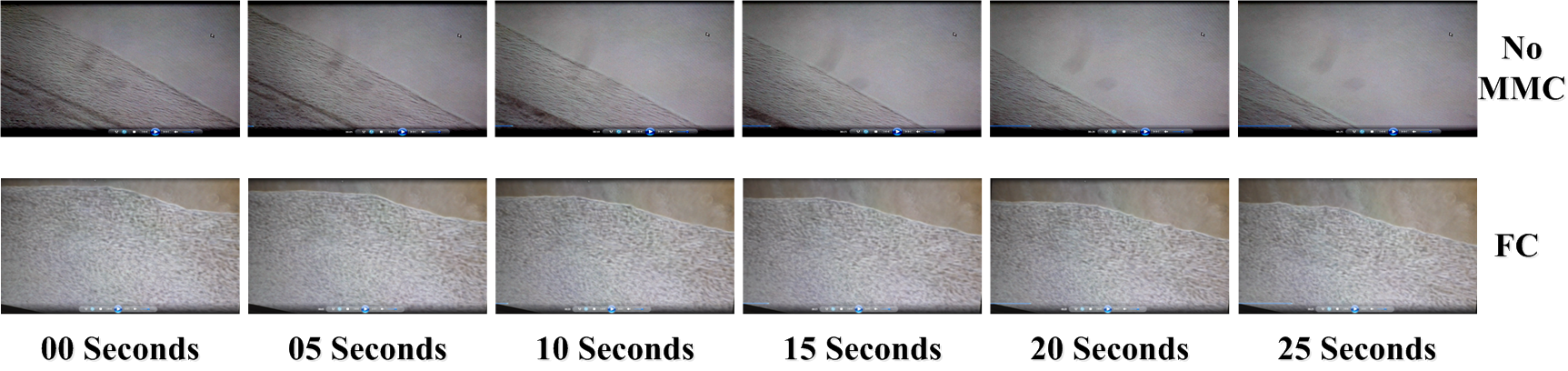


**Fig. S12.** AFM analysis of cell-layers produced after 6 days in culture further confirms the presence of high quarter staggered collagenous matrix in the crowded (FC) samples.

**
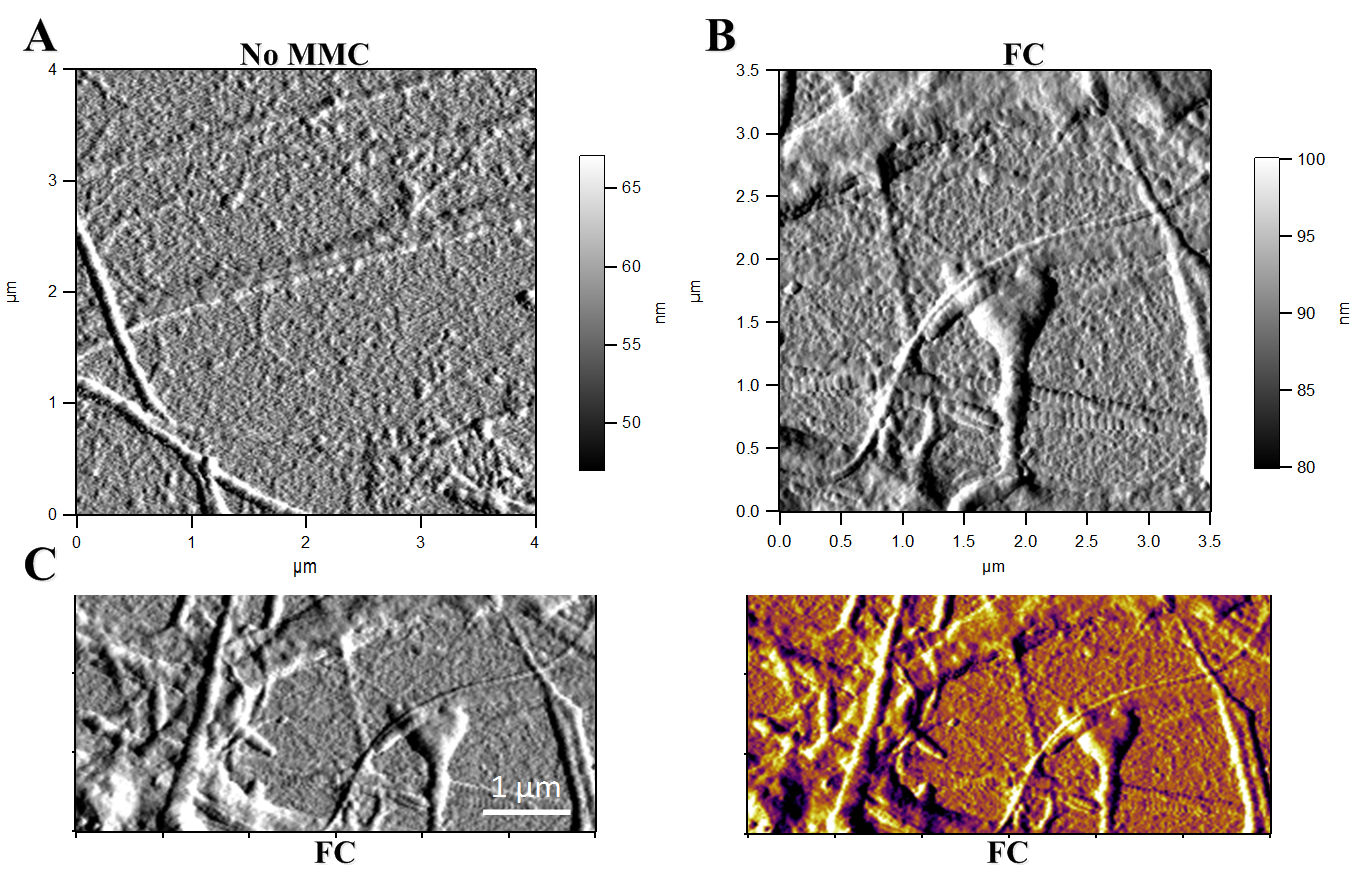
**

**Fig. S13.** Relative immunofluorescence intensity confirmed the high deposition of collagenous proteins (I, III, IV, V, VI) and glycoproteins (fibronectin) under MMC conditions (0.5 % HS, FC), whilst no difference was observed for unintended differentiation markers (*α* SMA) at all time points (2, 4 and 6 days).


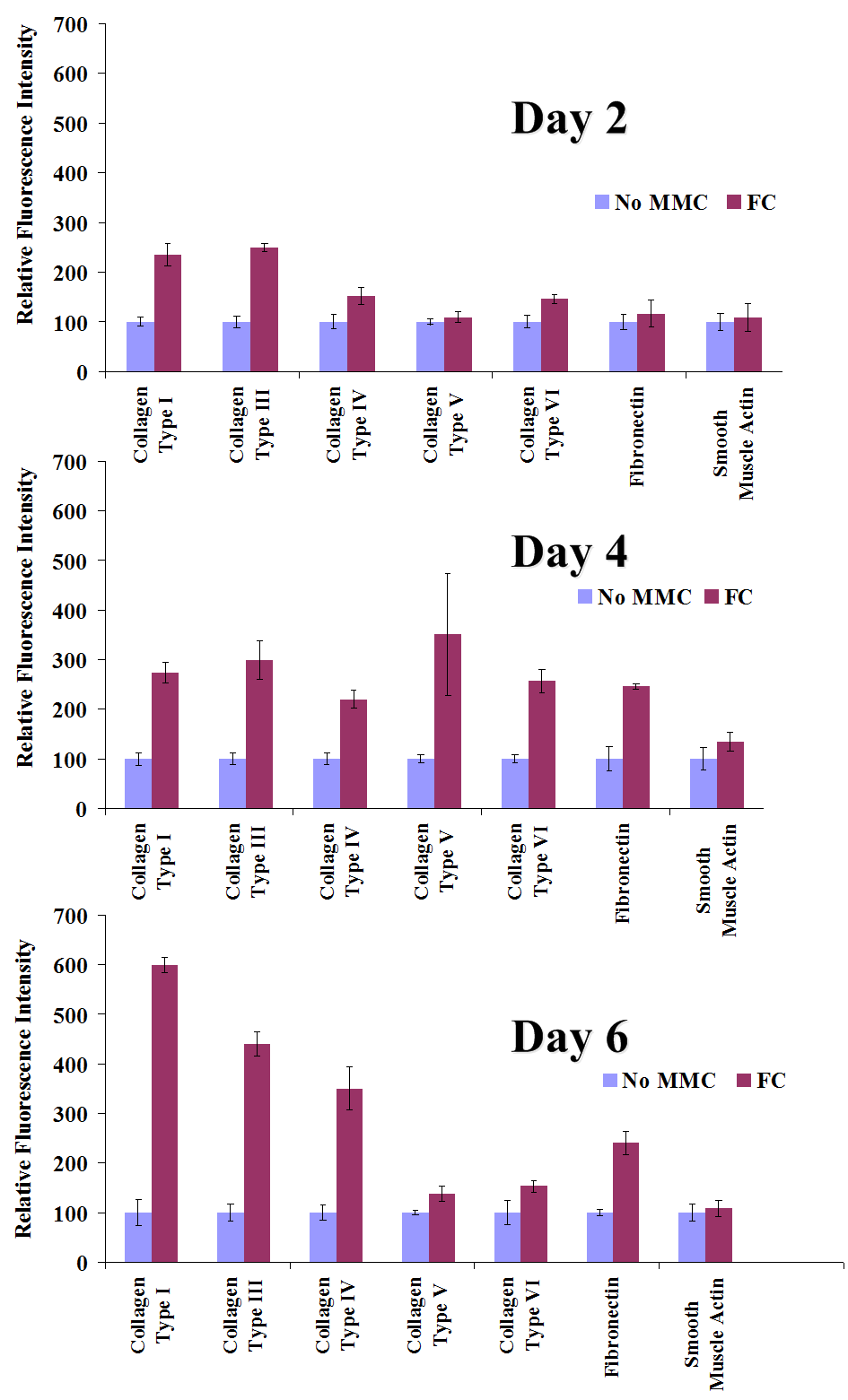


**Fig. S14.** Immunofluorescence images for CD34 and keratocan after 2, 4 and 6 days in culture, with and without FC, in the presence of 0.5 % HS. Our data indicate that the cells did not express these markers. HCFs cease expression of these markers rapidly in culture and after exposure to serum.


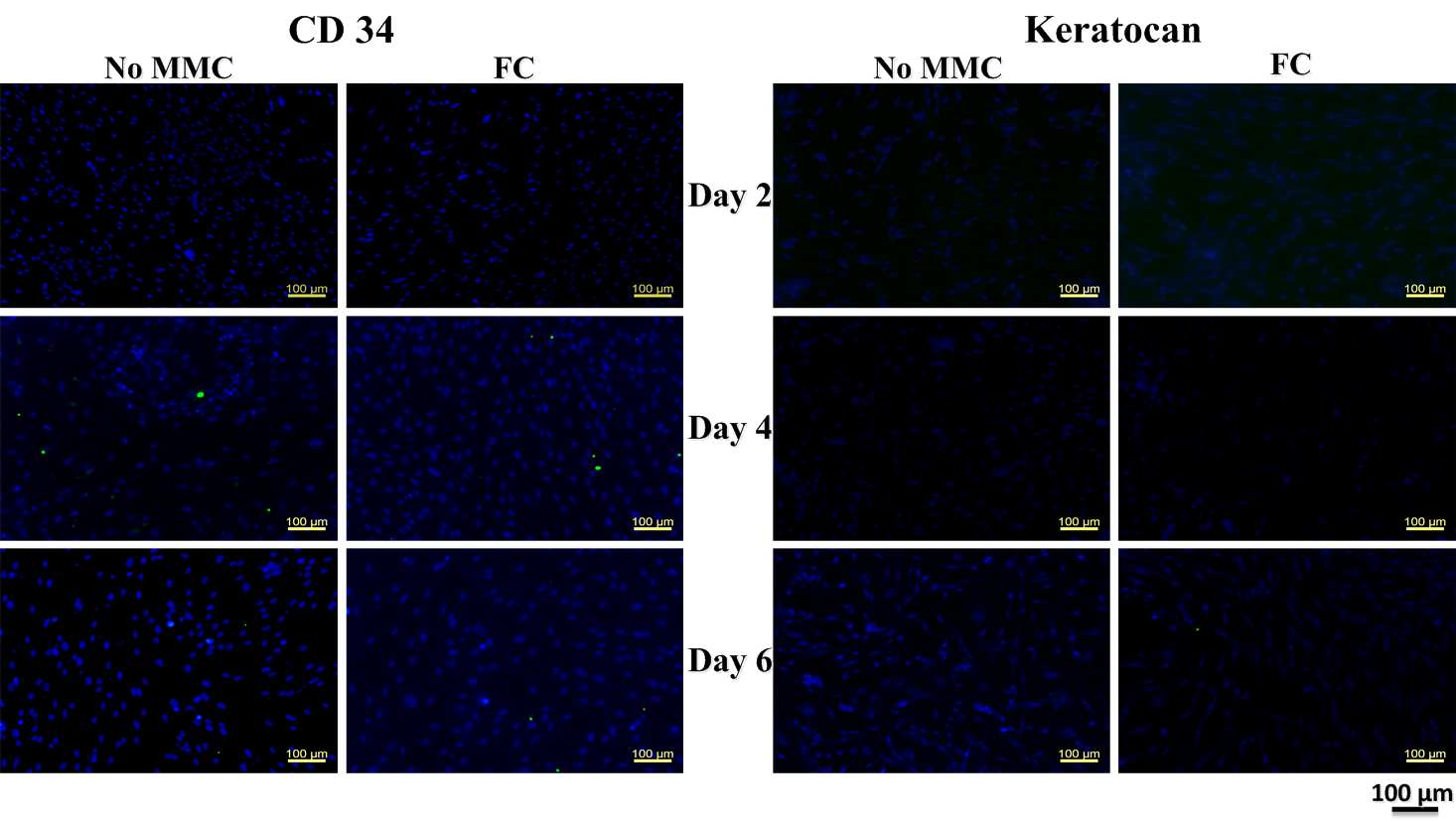

Supplement: Supplementary Information [file srep08729-s1.doc]
